# Hey surgeons! It is time to lead and be a champion in preventing and managing surgical infections!

**DOI:** 10.1186/s13017-020-00308-1

**Published:** 2020-04-19

**Authors:** Massimo Sartelli, Federico Coccolini, Fikri M. Abu-Zidan, Luca Ansaloni, Stefano Bartoli, Walter Biffl, Felice Borghi, Elie Chouillard, Yunfeng Cui, Rafael De Oliveira Nascimento, Belinda De Simone, Salomone Di Saverio, Therese Duane, Christian Eckmann, Hani O. Eid, Carlos Augusto Gomes, Felipe Couto Gomes, Andreas Hecker, Birgit Hecker, Arda Isik, Kamal M. F. Itani, Ari Leppaniemi, Andrey Litvin, Davide Luppi, Ronald Maier, Ramiro Manzano-Nunez, Sanjay Marwah, John Mazuski, Ernest Moore, Gennaro Perrone, Kemal Rasa, Ines Rubio, Robert Sawyer, Francesco M. Labricciosa, Fausto Catena

**Affiliations:** 1 Department of Surgery, Macerata Hospital, Macerata, Italy; 2 General, Emergency and Trauma Surgery Department, Pisa University Hospital, Pisa, Italy; 3 Department of Surgery, College of Medicine and Health Sciences, UAE University, Al-Ain, United Arab Emirates; 4 General Surgery Department, Bufalini Hospital, Cesena, Italy; 5 Department of Vascular Surgery, Sant’Eugenio Hospital, Rome, Italy; 6 Trauma Surgery Department, Scripps Memorial Hospital, La Jolla, CA USA; 7 Department of Surgery, General and Oncologic Surgery Unit, Santa Croce e Carle Hospital, Cuneo, Italy; 8 Department of Surgery, Poissy Saint Germain Medical Center, Poissy, France; 9 Department of Surgery, Tianjin Nankai Hospital, Nankai Clinical School of Medicine, Tianjin Medical University, Tianjin, China; 10 Cirurgia Geral Hospital Unimed Belo Horizonte, Belo Horizonte, Brazil; 11 Cambridge Colorectal Unit, Cambridge University Hospitals NHS Foundation Trust, Addenbrooke’s Hospital, Cambridge Biomedical Campus, Hills Road, Cambridge, UK; 12 Department of General Surgery, University Hospital of Varese, ASST SetteLaghi, RegioneLombardia, Varese, Italy; 13 Envision Healthcare, Dallas, TX USA; 14 Department of General, Visceral and Thoracic Surgery, Klinikum Hannoversch-Münden Academic Hospital of Goettingen University, Goettingen, Germany; 15 Department of Emergency Medicine, Mediclinic Middle East, Airport Road Hospital, Abu Dhabi, United Arab Emirates; 16 Department of Surgery, Hospital Universitário Terezinha de Jesus, Faculdade de Ciências Médicas e da Saúde de Juiz de Fora, Juiz de Fora, Brazil; 17 Cirurgia Geral Hospital Lifecenter Belo Horizonte, Belo Horizonte, Brazil; 18 Department of General and Thoracic Surgery, University Hospital Giessen, Giessen, Germany; 19 Department of Anaesthesiology and Intensive Care, Saint Josef Hospital, Giessen, Germany; 20 Department of General Surgery, Faculty of Medicine, Erzincan University, Erzincan, Turkey; 21 Department of Surgery, Boston University School of Medicine, Boston, MA USA; 22 Abdominal Center, University Hospital Meilahti, Helsinki, Finland; 23 Surgical Disciplines, Immanuel Kant Baltic Federal University/Regional Clinical Hospital, Kaliningrad, Russian Federation; 24 Department of General and Emergency Surgery, ASMN Reggio Emilia, Reggio Emilia, Italy; 25 Department of Surgery, University of Washington, Seattle, WA USA; 26 Clinical Research Center, Fundacion Valle del Lili, Cali, Colombia; 27 Department of Surgery, Post-Graduate Institute of Medical Sciences, Rohtak, India; 28 Department of Surgery, School of Medicine, Washington University, Saint Louis, USA; 29 Department of Surgery, University of Colorado, Denver Health Medical Center, Denver, CO USA; 30 Department of Emergency Surgery, Parma Maggiore Hospital, Parma, Italy; 31 Department of Surgery, Anadolu Medical Center, Kocaali, Turkey; 32 General Surgery Department, Colorectal Surgery Unit, La Paz University Hospital, Madrid, Spain; 33 Department of Surgery, Western Michigan University School of Medicine, Kalamazoo, MI USA; 34 Global Alliance for Infections in Surgery, Vila Nova de Gaia, Portugal

**Keywords:** Antibiotic therapy, Antimicrobial resistance, Surgical infections, Infection prevention and control

## Abstract

Appropriate measures of infection prevention and management are integral to optimal clinical practice and standards of care. Among surgeons, these measures are often over-looked. However, surgeons are at the forefront in preventing and managing infections. Surgeons are responsible for many of the processes of healthcare that impact the risk for surgical site infections and play a key role in their prevention. Surgeons are also at the forefront in managing patients with infections, who often need prompt source control and appropriate antibiotic therapy, and are directly responsible for their outcome. In this context, the direct leadership of surgeons in infection prevention and management is of utmost importance. In order to disseminate worldwide this message, the editorial has been translated into 9 different languages (Arabic, Chinese, French, German, Italian, Portuguese, Spanish, Russian, and Turkish).

## Background

### The challenge

In a book, by the surgeon Sherwin B. Nuland, on the history of Ignaz Philipp Semmelweis [1], the author refers to puerperal fever as the “doctors’ plague,” because these same doctors and medical students, who treated the patients, spread the infection by their hands. During the mid-nineteenth century, a disease characterized by pain, general malaise, and high fever, known as “puerperal fever,” literally decimated the new mothers hospitalized in the Vienna University hospital where Dr. Semmelweis worked. Without knowing the existence of bacteria (discovered by Louis Pasteur only in the second half of nineteenth century), he understood that the mortality rate could be reduced by doctors’ hand washing with chlorinated lime solution before every examination. Semmelweis’s observations conflicted with the established scientific and medical opinions of the time, and he was treated as an outcast. He is now known as “father of infection control.” Beginning with the discovery of penicillin by Alexander Fleming in the late 1920s, antibiotics have revolutionized the field of medicine [2].

They have saved millions of lives each year and have even been used prophylactically for the prevention of infectious diseases. However, bacteria have developed resistance to antibiotics, causing infections that are more serious because they are increasingly resistant to antibiotics. Using this perspective, current infections may be defined as the new “doctors’ plague,” because the same doctors through inappropriate use of antibiotics and inadequate infection prevention are contributing to the development and spread of antimicrobial resistance (AMR).

Surgeons in their clinical practice are at the forefront in preventing and managing infections. However, among surgeons, appropriate measures of infection prevention are often disregarded. The lack of awareness of these measures has marginalized surgeons from this fight. In many hospitals around the world, surgeons are not involved in the antimicrobial stewardship programs despite the fact that they are frequent prescribers of antibiotics both for prophylaxis and therapy. Furthermore, surgeons are often not involved in infection prevention teams, yet they are primarily responsible for preventing hospital-acquired infections, particularly surgical site infections. We submit that if surgeons around the world would participate in this global fight, they will be pivotal leaders in addressing this challenge.

In order to disseminate worldwide this message, the editorial has been translated into 9 different languages: Arabic, Chinese, French, German, Italian, Portuguese, Spanish, Russian, and Turkish (Additional files 1, 2, 3, 4, 5, 6, 7, 8 and 9).

## Main text

### The global threat of AMR

Improving patient safety in today’s hospitals worldwide requires a systematic approach to combat AMR and to prevent and treat infections appropriately. The two concepts go hand-in-hand [3]. AMR has emerged as one of the major public health problems of the twenty-first century. This has resulted in a public health crisis of international proportions, which threatens the practice of modern medicine, animal health, and food security [4].

The threat of AMR represents arguably the greatest patient safety challenge of our time. It has been widely reported that the world is on the cusp of a “post-antibiotic era,” with the growth in multidrug resistant bacteria raising the prospect that modern medicine will be increasingly unable to treat what are currently considered to be routine infections. AMR is a natural phenomenon that occurs as bacteria evolve. However, human activities have accelerated the pace at which bacteria develop and propagate resistance.

### Global initiative to fight AMR

Addressing the rising threat of AMR requires a holistic and multidisciplinary approach—referred to as One Health—because antibiotics used to treat various animal infectious diseases may be very similar to those used to treat humans [5].

Resistant bacteria arising in humans, animals, or the environment may spread from one to the other and from one country to another. AMR is not limited to geographic or zoologic borders [3]. Healthcare workers must play a central role in preventing the emergence and spread of AMR. Hospitalized patients often have multiple risk factors for acquisition of AMR. Acute care facilities are incubators for the development of AMR [6].

The intensity of care and the highly susceptible patient populations create an environment which facilitates both the emergence and transmission of resistant organisms.

### Appropriate use of antibiotics

Appropriate use of antibiotics is an integral part of optimal clinical practice. Antibiotics can be lifesaving when treating patients with bacterial infections. But they are often used inappropriately, specifically when unnecessary or when administered for excessive duration or without consideration of pharmacokinetic principles [7, 8]. The misuse of antibiotics is widely accepted as a major driver of some emerging infections (such as *C. difficile*), the selection of resistant pathogens in individual patients, and for the continued development of AMR globally [3].

### Prevention of surgical site infections (SSIs)

In 2017, the *Global Alliance for Infections in Surgery* shared with over 230 experts from 83 different countries a global declaration on appropriate use of antimicrobial agents in hospitals worldwide [3]. Within this declaration, the authors highlighted the contribution of antibiotic exposure, misuse, and overuse to the development of AMR and outlined the fundamental principles of appropriate antibiotic prophylaxis and therapy across the surgical pathway. Efforts to prevent hospital-acquired infections (HAIs) were not specifically highlighted in their declaration, but they are of significant importance in limiting antibiotic exposure.

Prevention is better than cure, and every infection prevented is one that needs no treatment. Prevention of infection can be cost effective and implemented everywhere, even where resources are limited. The surgical community continues to be cavalier in its approach to infection prevention and control. Patients with medical devices (central lines, urinary catheters, ventilators) or who undergo surgical procedures are at risk of acquiring HAIs. HAIs result in significant morbidity and mortality, prolong the duration of hospital stay, and necessitate additional diagnostic and therapeutic interventions. Surgeons continue to be obtuse to this reality with limited response to requests for intervention. Surgical site infections (SSIs) are the most common HAIs among surgical patients. In recent years, many comprehensive sets of guidelines for the prevention of surgical site infections have been published [9–11]. Despite clear evidences and guidelines to direct SSI prevention strategies, compliance is universally poor [12].

### Source control in surgical infections

When surgical infection occurs, the source of infection should be recognized and controlled. Whether related to a catheter, abscess or device all measures should be undertaken to eliminate the source and reduce the bacterial inoculum [13, 14]. Appropriate source control is of outmost importance in the management of surgical infections. Intra-abdominal infections along with soft tissues infections are the sites where source control is impactful. In these settings, an appropriate source control can improve patients’ outcome and reduce prolonged courses of antibiotic therapy. As a general principle, every verified source of infection should be controlled as soon as possible. The level of urgency of treatment is determined by the affected organ(s), the relative speed at which clinical symptoms progress, and the underlying physiological stability of the patient.

### Obstacles to overcome for surgeons

Leading international organizations acknowledge that collaboration is essential in providing care that is appropriate to meet the needs of patients, optimize individual health outcomes, and overall health care delivery [15]. A collaborative approach allows each member of the team to contribute expertise and to be responsible for their respective contributions to patient care. To be a champion in preventing and managing infections across the surgical spectrum involves the creation of a culture of collaboration in which infection prevention and control, antimicrobial stewardship, and optimal surgical approaches are all taken into consideration and respected by all members of the team.

Surgeons are at the forefront in preventing infections. Surgeons are responsible for many of the processes of healthcare that impact the risk for SSIs and play an important role in their prevention. Surgeons are also at the forefront in managing patients with infections, who often need prompt source control and adequate antibiotic therapy being directly responsible for their outcome. In this context, their leadership in the multidisciplinary efforts to improve the quality of the surgical patient is critical. To be leaders, surgeons should be aware that the appropriate prevention and management of infections across the surgical spectrum is an integral part of best practice.

In hospitals, cultural, contextual, and behavioral determinants influence clinical practice. Improving behavior in infection prevention and management remains a challenge. A range of factors such as diagnostic uncertainty, fear of clinical failure, time pressure, or organizational contexts can complicate the surgeons’ approach towards infections. However, due to cognitive dissonance (recognizing that an action is necessary but not implementing it), changing behavior is challenging. There are generally three primary levels that may influence surgeons’ behavior modification on infection prevention and management. These include the following:

1. Intrapersonal level.
2. Interpersonal level.
3. Institutional or organizational level.

On an individual level, surgeons should have the necessary knowledge, skills, and abilities to implement effective infection prevention and management practices. Improving their knowledge may influence their perceptions and motivate them to change behavior. Education and training represent an important component for accurate implementation of recommendations. Education of surgeons in prevention and management of infections should begin at the undergraduate level and be consolidated with further training throughout the postgraduate years. Hospitals are responsible for educating clinical staff. Education techniques, such as educational workshops, should be implemented in each hospital worldwide according to their own resources.

### Surgeons as leaders and champions in an interdisciplinary group to fight AMR

However, increasing knowledge alone may not be sufficient and may not be effective in changing practice, unless education is interactive and continuous, and includes discussions on evidence, local consensus, feedback on performance (by peers), making personal and group learning plans, etc. Identifying a local opinion leader to serve as a champion is important because the “champion” may integrate best clinical practices and drive their colleagues into changing behaviors. Surgeons with satisfactory knowledge in surgical infections may provide feedback to the prescribers and implement change within their own sphere of influence interacting directly with both the antimicrobial stewardship group and the infection control group. Excluding surgeons have increased the barriers to applying best practices.

Finally, organizational obstacles may influence infection prevention and management. Many different hospital disciplines are typically involved in infection prevention and management, making collaboration, coordination, communication, teamwork, and efficient care an essential component of success. There is now a substantial body of evidence that effective teamwork in health care contributes to improved outcomes. Using this approach reinforces, the concept that each discipline brings particular expertise and is responsible for their respective contributions to patient care. Across the surgical spectrum, it means creating a culture of collaboration in which infection prevention and control, antimicrobial stewardship, and correct surgical approach are all of utmost importance and properly coordinated. In this context, the direct leadership of surgeons, who are directly responsible for their patients, is of utmost importance.

## Conclusions

If surgeons around the world participate in this global fight, they will be pivotal leaders in addressing this challenge. Otherwise, they will be contributors to the worst crisis that the world health is facing.

Hey surgeons! It is your call! It is your time to participate and your time to lead. Now is the time to act!

## Supplementary information

**Additional file 1:.** Arabic translation.

**Additional file 2:.** Chinese translation.

**Additional file 3:.** French translation.

**Additional file 4:.** German translation.

**Additional file 5:.** Italian translation.

**Additional file 6:.** Portuguese translation.

**Additional file 7:.** Spanish translation

**Additional file 8:.** Russian translation.

**Additional file 9:.** Turkish translation.

## Data Availability

Not applicable.

## Abbreviations

AMR: Antimicrobial resistance
HAIs: Healthcare-associated infections
SSIs: Surgical site infections

## References

[1] Nuland SB (2004). The doctors’ plague: germs, childbed fever, and the strange story of Ignac Semmelweis.

[2] Alharbi SA, Wainwright M, Alahmadi TA, Salleeh HB, Faden AA, Chinnathambi A (2014). What if Fleming had not discovered penicillin?. Saudi J Biol Sci.

[3] Global Alliance for Infections in Surgery Working Group. A Global Declaration on Appropriate Use of Antimicrobial Agents across theSurgical Pathway. Surg Infect (Larchmt). 2017;18:846–53. 10.1089/sur.2017.219 29173054

[4] Venter H, Henningsen ML, Begg SL (2017). Antimicrobial resistance in healthcare, agriculture and the environment: the biochemistry behind the headlines. Essays Biochem.

[5] McEwen SA, Collignon PJ (2018). Antimicrobial resistance: a One Health Perspective. Microbiol Spectr.

[6] MacVane SH (2017). Antimicrobial resistance in the intensive care unit: a focus on gram-negative bacterial infections. J Intensive Care Med.

[7] Sartelli M, Weber DG, Ruppé E, Bassetti M, Wright BJ, Ansaloni L (2016). Antimicrobials: a global alliance for optimizing their rational use in intra-abdominal infections (AGORA). World J Emerg Surg.

[8] Sawyer RG, Claridge JA, Nathens AB, Rotstein OD, Duane TM, Evans HL (2015). Trial of short-course antimicrobial therapy for intraabdominal infection. NEJM.

[9] WHO - World Health Organization. WHO Global Guidelines 2016 for the prevention of surgical site infection. 2016. http://www.who.int/gpsc/ssi-guidelines/en/. Accessed 09 Mar 2020.

[10] Berríos-Torres SI (2016). Evidence-based update to the U.S. Centers for Disease Control and Prevention and Healthcare Infection Control Practices Advisory Committee guideline for the prevention of surgical site infection: developmental process. Surg Infect (Larchmt).

[11] Ban KA, Minei JP, Laronga C, Harbrecht BG, Jensen EH, Fry DE (2017). American College of Surgeons and Surgical Infection Society: surgical site infection guidelines, 2016 update. J Am Coll Surg.

[12] Leaper DJ, Tanner J, Kiernan M, Assadian O, Edmiston CE (2015). Surgical site infection: poor compliance with guidelines and care bundles. Int Wound J.

[13] Marshall JC (2010). Principles of source control in the early management of sepsis. Curr Infect Dis Rev.

[14] Marshall JC, al Naqbi A (2009). Principles of source control in the management of sepsis. Crit Care Clin.

[15] WHO - World Health Organization. Implementation manual to support the prevention of surgical site infections at the facility level – Turning recommendations into practice. https://www.who.int/infection-prevention/publications/implementation-manual-prevention-surgical-site-infections.pdf. Accessed 9 Mar 2020.

