# Supplementary material for: Hey surgeons! It is time to lead and be a champion in preventing and managing surgical infections!
Source: World J Emerg Surg. 2020 Apr 19;15:28. doi: 10.1186/s13017-020-00308-1 (PMC7168830; doi:10.1186/s13017-020-00308-1)
Supplement: Supplementary file 1 — Additional file 1:. Arabic translation. [file 13017_2020_308_MOESM1_ESM.docx]

**Additional file 1.** Arabic translation.

By Hani O. Eid and Fikri M. Abu-Zidan.

**أيها الجراحون! لقد حان الوقت للقيادة كي نكون أبطالا في وقاية وعلاج الأخماج الجراحية!**

الخلاصة

**تعد التدابير المناسبة للوقاية من الأخماج وعلاجها جزءاً لا يتجزأ من معايير الرعاية والممارسة السريرية المثلى. فغالباً ما يتم التساهل في هذه التدابير فيما بين الجراحين. بالرغم من ذلك، فالجراحون هم سباقون في وقاية وعلاج الأخماج. فهم مسؤولون عن العديد من عمليات الرعاية الصحية التي تؤثر بشكل رئيسي على** *أخماج الموضع الجراحي، وهم يلعبون دوراً رئيسياُ في الوقاية منها. والجراحون هم أيضا في طليعة علاج المرضى الذين يعانون من الأخماج، الذين غالبا ما يحتاجون إلى السيطرة الفورية على مصدر العدوى والعلاج بالمضادات الحيوية المناسبة، وهم مسؤولون بشكل مباشر عن نتائجهم. وفي هذا السياق، فإن القيادة المباشرة للجراحين في الوقاية من الأخماج وعلاجها ذات أهمية قصوى.*

التحدي

في كتاب، من قبل الجراح شيروين ب. نولاند، على تاريخ إغناز فيليب سيملويس [1]، يشير المؤلف إلى حمى النفاس باسم "طاعون الأطباء"، لأن هؤلاء الأطباء وطلاب الطب، الذين عالجوا المرضى، هم الذين نشروا العدوى بأيديهم. فخلال منتصف القرن التاسع عشر، أدى مرض يتميز بالألم والإعياء العام والحمى المرتفعة، والمعروف باسم "حمى النفاس" إلى هلاك الأمهات الجدد اللائي أدخلن لمستشفى جامعة فيينا حيث يعمل الطبيب سيميلويس. فقد أدرك دون معرفته بوجود البكتيريا (التي اكتشفها لويس باستور في النصف الثاني من القرن التاسع عشر فقط)، أنه يمكن خفض معدل الوفيات عن طريق غسل يد الأطباء قبل كل فحص للمريض بمحلول الجير المكلور. وتضاربت ملاحظات سيمللويس مع الآراء العلمية والطبية الراسخة في ذلك الوقت. وهو يعرف الآن ب "أب مكافحة الأخماج".

أحدثت المضادات الحيوية ثورة في مجال الطب بدءا من اكتشاف ألكسندر فليمنغ للبنسلين في أواخر العشرينات من القرن العشرين. فقد أنقذت ملايين الأرواح كل عام، بل واستخدمت على نحو وقائي للوقاية من الأمراض المعدية. وبالرغم من ذلك، طورت الجراثيم مقاومتها للمضادات الحيوية، مما تسبب في أخماج أكثر خطورة لأنها مقاومة بشكل متزايد للمضادات الحيوية.

من هذا المنظور، يمكن تعريف الأخماج الحالية بأنها "طاعون الأطباء" الجديد، لأن الأطباء أنفسهم من خلال الاستخدام غير المناسب للمضادات الحيوية ومن خلال الوقاية غير الكافية من العدوى يساهمون في تطور وانتشار المقاومة لمضادات الجراثيم (AMR).

**يحتل الجراحون في ممارستهم السريرية مكان الصدارة في الوقاية من الأخماج وعلاجها. ومع ذلك، فغالباً ما يتجاهلون التدابير المناسبة للوقاية من الأخماج. وأدى عدم الوعي بهذه التدابير إلى تهميش الجراحين في هذه المعركة. ففي العديد من المستشفيات حول العالم، لا يتم اشراك الجراحين في برامج** إدارة مضادات الجراثيم على الرغم من أنهم يصفون بشكل متكرر المضادات الحيوية لكل من الوقاية والعلاج. علاوة على ذلك، لا يشارك الجراحون في كثير من الأحيان في فرق الوقاية من العدوى. ومع ذلك، فهم يتحملون المسؤولية الأولى عن الوقاية من الإصابات المكتسبة في المستشفيات، وخاصة أخماج الموضع الجراحي. إننا نؤكد أنه إذا شارك الجراحون حول العالم في هذه المعركة العالمية، فسيكونون قادة محوريين في مواجهة هذا التحدي.

التهديد العالمي لمقاومة مضادات الجراثيم

**يتطلب تحسين سلامة المرضى في مستشفيات اليوم في جميع أنحاء العالم مقاربة منهجية لمكافحة مقاومة مضادات الجراثيم** AMR **للوقاية من الأخماج وعلاجها بشكل مناسب.** فهما يسيران جنبا إلى جنب [2].

لقد برزت **مقاومة مضادات الجراثيم** AMR كواحدة من أكبر مشكلات الصحة العامة في القرن الحادي والعشرين. وقد أدى ذلك إلى أزمة صحية عامة ذات أبعاد دولية، مما يهدد ممارسة الطب الحديث وصحة الحيوان والأمن الغذائي. ويمكن القول إن تهديد مقاومة مضادات الجراثيم AMR **يعد من أعظم تحديات سلامة المرضى في عصرنا.** وقد تعارف على نطاق واسع أن العالم على أعتاب "عصر ما بعد المضادات الحيوية"**، فتنامي الجراثيم المقاومة للأدوية المتعددة يزيد من احتمالية أن يكون الطب الحديث عاجزاً** على نحو متزايد **على علاج ما يعتبر حالياً عدوى عادية.** فمقاومة مضادات الجراثيم AMR **تعد ظاهرة طبيعية تحدث مع تطور الجراثيم. ومع ذلك، فإن الأنشطة البشرية قد سارعت من وتيرة تطور الجراثيم وانتشار مقاومة مضادات الجراثيم**

المبادرة العالمية لمكافحة مقاومة مضادات الجراثيم  **AMR**

يتطلب التصدي للتهديد المتزايد ل**مقاومة مضادات الجراثيم** AMR نهجاً شاملاً ومتعدد التخصصات ــ يشار إليه باسم "صحة واحدة " - لأن المضادات الحيوية المستخدمة لعلاج مختلف الأمراض الحيوانية المعدية قد تكون مماثلة لتلك المستخدمة للبشر. قد تنتشر الجراثيم المقاومة التي تنشأ في البشر أو الحيوانات أو البيئة من وسيط إلى آخر، ومن بلد إلى آخر. فلا تقتصر **مقاومة مضادات الجراثيم** AMR على الحدود الجغرافية أو الحيوانية [2]. وبالتالي، يلعب العاملون في الرعاية الصحية دوراً رئيسياً في منع ظهور **مقاومة مضادات الجراثيم** AMR وانتشارها.

**غالبا ما يكون لدى مرضى المستشفيات عوامل خطر متعددة لاكتساب مقاومة مضادات الجراثيم** AMR**. فمرافق الرعاية الحادة، هي حاضنات لتطوير مقاومة مضادات الجراثيم** AMR**. وكثافة الرعاية والأشخاص الأكثر عرضة للإصابة يخلقان بيئة مناسبة تسهل ظهور وانتقال الجراثيم المقاومة.**

الاستخدام المناسب للمضادات الحيوية

**الاستخدام المناسب للمضادات الحيوية هو جزء لا يتجزأ من الممارسة السريرية المثلى. فيمكن أن تكون المضادات الحيوية منقذة للحياة عند علاج المرضى الذين يعانون من الالتهابات الجرثومية. لكن غالبًا ما يتم استخدامها بشكل غير مناسب، وتحديداً عندما لا تكون ضرورية أو عندما تعطى لمدة مفرطة أو بدون مراعاة مبادئ الحركة الدوائية في الجسم [3-4]. يمكننا أن نقبل أن إساءة استخدام المضادات الحيوية هو المحرك الرئيسي لبعض الأخماج الناشئة (مثل المطثية العسيرة)، وانتقاء مسببات الأمراض المقاومة لدى المرضى بشكل فردي، واستمرار تطوير مقاومة مضادات الجراثيم** AMR **على الصعيد العالمي. بالإضافة إلى ذلك، اشارت الدراسات الحديثة للدور الرئيسي للوسط المعوي في الأمراض الحادة والمزمنة، وقابليته للتأثر بالمضادات الحيوية الغير ملائمة.**

الوقاية من أخماج الموضع الجراحي

**شارك في عام 2017 في التحالف العالمي للأخماج الجراحية أكثر من 230 خبيراً من 83 دولة مختلفة لإصدار اعلاناً عالمياً بشأن الاستخدام المناسب لعوامل مضادات الجراثيم في المستشفيات في جميع أنحاء العالم [1]. سلط المؤلفون ضمن هذا الإعلان الضوء على مساهمة التعرض للمضادات الحيوية وإساءة استخدامها والإفراط في استخدامها في نشوء مقاومة مضادات الجراثيم** AMR**، وحددوا المبادئ الأساسية للوقاية والعلاج باستخدام المضادات الحيوية المناسبة عبر المسار الجراحي.**

**لم يتم تسليط الضوء في إعلانهم بشكل خاص على الجهود المبذولة لمنع الأخماج المكتسبة من المستشفيات على وجه التحديد مع كونها ذات أهمية قصوى في الحد من التعرض للمضادات الحيوية.**

**الوقاية خير من العلاج، وكل عدوى يتم الوقاية منها لا تحتاج إلى علاج. يمكن أن تكون الوقاية من العدوى فعالة من حيث التكلفة ويتم تنفيذها في كل مكان، حتى عندما تكون الموارد الاقتصادية محدودة.**

**لا يزال المجتمع الجراحي متردداً في نهجه للوقاية من الأخماج والسيطرة عليها. فالمرضى الذين يحتاجون الأدوات الطبية (كالقساطر الوريدية المركزية، والقساطر البولية، وأجهزة التنفس الصناعي) أو الذين يخضعون لإجراءات جراحية معرضون لخطر الإصابة بالأخماج المكتسبة من المستشفيات. حيث تؤدي الأخماج المكتسبة من المستشفيات إلى ارتفاع معدلات الاختلاطات والوفيات، وإطالة مدة الإقامة في المستشفى، مما يتطلب تدخلات تشخيصية وعلاجية إضافية. يستمر الجراحون في إهمال هذا الواقع باستجابتهم المحدودة للحاجة إلى التدخل.**

**تعد أخماج الموضع الجراحي أكثر أنواع الأخماج المكتسبة من المستشفيات شيوعاً بين المرضى الخاضعين لعمليات جراحية. فقد تم في السنوات الأخيرة نشر العديد من** *القواعد الارشادية* **الشاملة للوقاية من أخماج الموضع الجراحي [5-7]. وعلى الرغم من توافر الأدلة و***القواعد الارشادية* **الواضحة لتوجيه استراتيجيات الوقاية من أخماج الموضع الجراحي، فإن اتباع هذه القواعد ضعيف على مستوى العالمي.**

السيطرة على مصدر الأخماج الجراحية

**عند حدوث الخمج الجراحي، يجب التعرف على مصدر الخمج والسيطرة عليه. فسواء كان ذلك مرتبطاً بالقسطرة أو الخراج أو الجهاز، يجب اتخاذ جميع التدابير للقضاء على المصدر وتقليل التكاثر الجرثومي [8-9]. فالسيطرة المناسبة على المصدر لها أهمية قصوى في علاج الأخماج الجراحية. فالأخماج داخل البطن إلى جانب أخماج الأنسجة الرخوة هي الاماكن التي يكون فيها السيطرة على مصدر الخمج ناجعاً. ففي هذه الحالات، يمكن للتحكم المناسب بمصدر الخمج أن يحسن من نتائج المرضى ويقلل من فترة استخدام المضادات الحيوية. كقاعدة عامة، يجب السيطرة سريعاً على كل مصدر** من مصادر **الأخماج** التي تم التحقق **منها. ويتم تحديد مستوى سرعة العلاج من خلال العضو (أو الأعضاء) المتأثرة، والسرعة النسبية التي تتقدم بها الأعراض السريرية، والاستقرار الفسيولوجي الأساسي للمريض.**

العقبات التي يجب على الجراحين التغلب عليها

**تقر المنظمات الدولية الرائدة بأن التعاون ضروري لتوفير الرعاية المناسبة لتلبية احتياجات المرضى، وتحسين النتائج الصحية الفردية وتقديم الرعاية الصحية العامة [10].**

**يسمح النهج التعاوني لكل عضو من أعضاء الفريق بالمساهمة بخبراته ومسؤولياته في رعاية المرضى. يتطلب كي تكون بطلاً في الوقاية والعلاج من الأخماج عبر الطيف الجراحي، خلق ثقافة تعاون يتم فيها مراعاة واحترام جميع أعضاء الفريق المساهم في الوقاية من الأخماج والسيطرة عليها وإدارة مضادات الجراثيم باتباع النهج الجراحي الصحيح.**

**الجراحون هم السباقون في الوقاية من الأخماج. فالجراحون مسؤولون عن العديد من عمليات الرعاية الصحية التي تؤثر على خطر أخماج المواضع الجراحية، ويلعبون دوراً مهماً في الوقاية منها. والجراحون هم أيضاً في طليعة علاج المرضى الذين يعانون من الأخماج، والذين غالباً ما يحتاجون إلى سيطرة فورية على مصادر الأخماج وعلاجها بالمضادات الحيوية الملائمة لكونهم مسؤولين بشكل مباشر عن نتائجهم. وفي هذا السياق، تعتبر قيادتهم للجهود متعددة التخصصات لتحسين جودة المريض الجراحي أمراً بالغ الأهمية.**

لكي نكون قادة، يجب أن يدرك الجراحون أن الوقاية والعلاج المناسب للأخماج عبر الطيف الجراحي جزء لا يتجزأ من الممارسات الطبية ذات المستوى العالي.

تؤثر المحددات الثقافية والسياقية والسلوكية على الممارسة السريرية في المستشفيات. ولا يزال تحسين السلوك في الوقاية من الأخماج وعلاجها تحديا اساسياً.

هناك مجموعة من العوامل مثل عدم اليقين التشخيصي والخوف من الفشل السريري وضيق الوقت والحواجز التنظيمية والتي تعقد نهج الجراحين تجاه الأخماج. إن تغيير السلوك النمطي يعد تحدياً في الممارسة السريرية وهو ما يعرف بالتنافر المعرفي (إدراك أن الإجراء ضروري ولكن لا يتم تنفيذه).

عموماً هنالك ثلاثة مستويات أساسية قد تؤثر على تعديل سلوك الجراحين في الوقاية من الأخماج وعلاجها. وهذه تشمل:

1. **المستوى الفردي**
2. **المستوى الشخصي**
3. **المستوى المؤسسي أو التنظيمي**

**على المستوى الفردي، يجب أن يكون لدى الجراحين المعرفة والمهارات والقدرات اللازمة للممارسة الفعالة للوقاية من الأخماج وعلاجها. فقد يؤثر تحسين معرفتهم على تصوراتهم ويحفزهم على تغيير سلوكهم. كما يمثل التعليم والتدريب مكوناً مهماً للتطبيق الدقيق للتوصيات. فيجب أن يبدأ تعليم الجراحين على أسس الوقاية من الأخماج وعلاجها على المستوى الجامعي** ويعزز بمزيد من التدريب طوال سنوات الدراسات العليا.

الجراحين كقادة وأبطال في مجموعة متعددة التخصصات لمحاربة مقاومة مضادات الجراثيم **AMR**

**إن زيادة المعرفة وحدها قد لا تكون كافية وقد لا تكون فعالة في تغيير الممارسة، ما لم يكن التعليم تفاعلياً ومستمراً، ويشمل مناقشات حول الأدلة العلمية، والإجماع المحلي، والتغذية الاسترجاعية على الأداء (من قبل أقرانهم)، ووضع خطط التعلم الشخصية والجماعية،** وما إلى ذلك. **فمن المهم تحديد قائد محلي ذي رأي سديد ليكون بمثابة بطل لأن "البطل" قد يدمج أفضل الممارسات السريرية ويدفع زملائه إلى تغيير سلوكياتهم. وقد يوفر الجراحون الذين لديهم معرفة مناسبة في الأخماج الجراحية تغذية استرجاعية للواصفين، وينفذون التغيير في محيط نفوذهم بالتفاعل المباشر مع كل من مجموعة** إدارة مضادات الجراثيم **ومجموعة السيطرة على الأخماج. إن استبعاد الجراحين زاد الحواجز أمام تطبيق أفضل الممارسات السرسرية.**

**أخيراً، قد تؤثر العوائق التنظيمية على الوقاية من الأخماج وعلاجها. و**عادة ما تشارك العديد من التخصصات المختلفة في المستشفيات في الوقاية **من الأخماج وعلاجها، مما يجعل التعاون والتنسيق والتواصل والعمل الجماعي والرعاية الفعالة عنصراً أساسياً في النجاح. يوجد الآن مجموعة كبيرة من الأدلة على أن العمل الجماعي الفعال في مجال الرعاية الصحية يساهم في تحسين النتائج. إن استخدام هذا النهج يعزز المفهوم القائل بأن كل تخصص يجلب خبرة خاصة ويكون مسؤولاً عن مساهماته في رعاية المرضى. فع**بر الطيف الجراحي، يمكن خلق ثقافة التعاون التي تكون فيها الوقاية من الأخماج ومكافحتها، والإشراف على **مجموعة** إدارة مضادات الجراثيم، والنهج الجراحي الصحيح كلها ذات أهمية قصوى ومنسقة بشكل صحيح. وفي هذا السياق، فإن القيادة المباشرة للجراحين، المسؤولين مباشرة عن مرضاهم، ذات أهمية قصوى.

**الاستنتاجات**

إذا شارك الجراحون في جميع أنحاء العالم في هذه المعركة العالمية، فسيكونون قادة محوريين في مواجهة هذا التحدي. وإلا، فسيكونون مشاركين في أسوأ أزمة تواجهها الصحة العالمية.

أيها الجراحون! انه دوركم! لقد حان الوقت للمشاركة وتولي القيادة. لقد حان وقت العمل!
