# Supplementary material for: Hey surgeons! It is time to lead and be a champion in preventing and managing surgical infections!
Source: World J Emerg Surg. 2020 Apr 19;15:28. doi: 10.1186/s13017-020-00308-1 (PMC7168830; doi:10.1186/s13017-020-00308-1)
Supplement: Supplementary file 2 — Additional file 2:. Chinese translation. [file 13017_2020_308_MOESM2_ESM.docx]

**Additional file 2.** Chinese translation.

By Yunfeng Cui.

**大家好,外科医生们!现在是时候在预防和控制外科感染方面发挥领导作用，并成为领军人物了!**

**摘要**

合理的感染预防和处理措施是优化临床实践和护理的重要组成部分。在外科医生当中，这些措施常常被忽视。然而，外科医生又恰恰在预防和控制感染方面处于最前沿。外科医生负责许多具有手术部位感染风险的医疗过程，并在预防过程中扮演关键作用。外科医生在治疗感染患者方面也处于前沿，并直接对治疗结果负责，病人通常需要及时的控制感染源和适当的抗生素治疗。在这种情况下，外科医生在感染预防和管理方面的领导力是至关重要的。

**面临的挑战**

在外科医生舍温•纽兰所著的《伊格纳兹•菲利普•塞梅尔维斯•的历史》[1]一书中，作者将产褥热称为“医生的瘟疫”，因为正是这些医生和医科学生在治疗病人时，通过双手传播了感染。19世纪中期，一种以疼痛、全身不适和高热为特征的疾病，即所谓的“产褥热”，大量杀死了在维也纳大学医院( 塞梅尔维斯医生工作的地方)住院的新妈妈们。他当时并不知道细菌的存在(直到19世纪下半叶才被路易斯•巴斯德发现)，只知道医生在每次检查前用氯化石灰溶液洗手可以降低死亡率。塞梅尔维斯医生的观察与当时的科学和医学观点相冲突。但如今，他被称为“感染控制之父”。

从20世纪20年代晚期亚历山大•弗莱明发现青霉素开始，抗生素就彻底改变了医学领域。它们每年拯救数百万人的生命，甚至被用于预防感染性疾病。

然而，正如简单达尔文进化论所预测的那样，细菌已经产生了对抗生素的耐药性，导致了更严重的感染，因为它们对抗生素的耐药性越来越强。

从这一角度看，如今的感染可被定义为新的“医生的瘟疫”。因为正是这些医生通过不适当使用抗生素和不充分的感染预防，促进着抗菌素耐药性(AMR)的发展和传播。

外科医生在其临床实践中处于感染预防和处理的前沿。然而，在外科医生当中，适当的感染预防措施却常常被忽视。由于缺乏对这些措施的认识，外科医生在这场斗争中被边缘化。在世界各地的许多医院，外科医生并没有参与抗菌药物管理项目。尽管出于预防和治疗的目的，他们需要经常开抗生素处方。此外，外科医生通常不参与感染预防小组，但他们却主要负责预防医院获得性感染，特别是手术部位感染。

我们认为，如果全世界的外科医生都参与到这场全球战斗中来，他们将成为应对这一挑战的领军人物。

**抗菌素耐药性(AMR)的全球威胁**

改善当今世界各地医院的患者安全，需要一种系统的方法来对抗抗生素耐药，并适当地预防和治疗感染。这两者是相辅相成的[2]。

抗生素耐药已成为21世纪主要的公共卫生问题之一。这导致了一场国际范围的公共卫生危机，威胁到现代医学实践、动物健康和粮食安全。抗生素耐药性的威胁可以说是我们这个时代病人安全所面临的最大挑战。据广泛报道，世界正处于后抗生素时代的风口浪尖上，随着耐多药细菌的增长，现代医学将越来越无法治疗目前被认为是常规的感染疾病，这种预期愈发显著。抗生素耐药性是细菌进化过程中出现的一种自然现象。然而，人类活动加速了细菌产生和传播耐药性的速度。

**抗击抗生素耐药性的全球倡议**

应对抗生素耐药性日益严重的威胁，需要一种被称为“同一健康”的全面多学科的方法，因为用于治疗各种动物感染疾病的抗生素可能与用于人类的抗生素类似。在人类、动物或环境中产生的耐药细菌可能从一个传播到另一个，并从一个国家传播到另一个国家。抗菌素耐药性并不局限于地理或动物边界[2]。因此，卫生保健工作者在预防抗菌素耐药性的出现和传播方面发挥了中心作用。

住院患者在抗菌素耐药性的发生上常常存在多种危险因素。急性护理设施是抗菌素耐药性发展的孵化器。护理的强度和高度易感人群创造了促进耐药生物的出现和传播的环境。

**抗生素合理使用 - 外科医生应对抗生素耐药性的目标1**

合理使用抗生素是最佳临床实践的一个必要部分。在治疗细菌感染患者时，抗生素可以挽救生命。但它们的使用往往不恰当，特别是在不必要或使用时间过长或未考虑药代动力学原则的情况下[3-4]。广泛认为抗生素的滥用是某些新发感染(如艰难梭菌)、个别患者耐药病原体的筛选及全球抗生素耐药性持续发展的主要驱动因素。此外，最近的研究还指出肠道菌群在急慢性疾病中的关键作用，以及对不适当抗生素应用的脆弱性。

**预防手术部位感染(SSI) - 外科医生应对抗生素耐药性的目标2**

2017年，全球外科感染联盟与来自83个不同国家的230多名专家分享了关于在全球医院合理使用抗微生物药物的全球宣言[1]。在这份声明中，作者强调了抗生素暴露、误用和过度使用对抗生素耐药性发展的作用，并概述了外科领域抗生素预防和治疗合理应用的基本原则。

他们的宣言没有特别强调预防医院获得性感染(HAIs)措施，但它们在限制抗生素接触方面具有重要意义。

预防胜于治疗，每一种预防的感染不需要治疗。在任何地方，即使在资源有限的地方，预防感染都是具有成本效益和可实施的。

外科界在预防和控制感染方面仍然漫不经心。使用医疗设备(中央管道、导尿管、呼吸机)或接受外科手术的病人有医院获得性感染的风险。医院获得性感染导致了显著的发病率和死亡率，延长了住院时间，并且需要额外的诊断和治疗干预。但外科医生仍然对这一现实感到迟钝，对干预的反应有限。

手术部位感染(SSIs)是外科患者中最常见的医院获得性感染。近年来，许多综合性的外科手术部位感染预防指南已经出版[5-7]。尽管有明确的证据和指导方针来指导手术部位感染的预防策略，但依从性普遍较差。

**外科感染的早期源头控制 - 外科医生应对抗生素耐药性的目标3**

当发生外科感染时，应识别和控制感染源。无论是和导管、脓肿或器械相关，都应采取一切措施消除感染源，减少细菌接种[8-9]。适当的感染源控制在外科感染的处理中是最重要的。腹腔内感染和软组织感染是源头控制的有效部位。在这些情况下，适当的感染源控制可以改善患者的预后，减少抗生素治疗的疗程。作为一般原则是，应尽快控制每一个经核实的感染源。治疗的紧迫程度由受影响器官、临床症状进展的相对速度和患者潜在的生理稳定性决定。

**外科医生要克服的障碍**

主流国际组织认识到，在提供满足患者需求的医疗、优化个体医疗结果和整体卫生保健服务方面，多方合作是至关重要的[10]。

相互协作的方法可以使团队中每个成员贡献专业知识，并对各自的患者医疗护理工作负责。要成为预防和处理外科感染的领军人物，需要有一种相互协作的氛围，在这种环境中，所有团队成员都需要考虑并重视感染预防和控制、抗菌药物管理和正确手术方法的选择。

外科医生处在感染预防的前沿。外科医生涉及许多影响手术部位感染风险的医疗过程，并在预防过程中发挥重要作用。外科医生处于感染患者处理的最前沿，感染患者往往需要及时感染源控制和充分的抗生素治疗，这直接关系到他们的预后。在这种背景下，他们在多学科协作中的领导力可以改善外科危重病人的救治质量。

作为领导者，外科医生应该意识到，整个外科范围内适当的感染预防和感染管理是良好医疗实践的必要组成部分。

在医院，文化、环境和行为决定因素影响临床实践。改善感染预防和管理的行为仍然是一个挑战。

诊断的不确定性、对临床失败的恐惧、时间压力或组织环境等一系列因素可能会使外科医生处理感染的方法复杂化。然而，由于认知的失调(认识到一个行动是必要的，但没有执行它)，行为改变依旧具有挑战性。

在感染预防和管理中主要有三个层面影响外科医生的行为。这些包括: 1)个人层面，2)人际层面，3)机构或组织层面。

在个人层面上，外科医生应具备必要的知识、技能和能力来实施有效的感染预防和管理措施。提高他们的知识会影响他们的感知，并激励他们改变行为。教育和培训是准确执行各项建议的重要组成部分。外科医生预防和控制感染的教育应从本科阶段开始，并在研究生阶段加强培训。医院负责教育临床工作人员。技能培训，如教育讲习班，应根据资源情况在世界各地的各个医院开展。

**在抗击抗生素耐药的跨学科的团队中外科医生是领导者和先行者**

然而，仅仅增加知识储备可能是不够的，在变化的实践中也可能是无效的，除非教育是互动的、持续的，包括证据讨论、地方共识、实践反馈(来自同行)、制定个人和团队学习计划等。要确定一个局部的观点，作为先行者的领导者是很重要的，因为先行者可以整合最好的临床实践，并推动他们的同事改变工作行为。在外科感染方面有经验的外科医生可以向药物处方者提供反馈，并在其自身的影响范围内实施改变，可以与抗菌药物管理组和感染控制组直接相互交流，需要除外外科医生增加进行良好临床实践的障碍。

最后，组织机构障碍可能影响感染预防和管理。许多不同的医院规定通常涉及感染预防和管理，协作、协调、沟通、团队合作和有效的护理是成功的重要组成部分。现在有大量证据表明，卫生保健中有效的团队合作有助于改善结果。通过这种方法，可以强化一个理念，每个学科具有特定的专业知识，并分别负责各自的病人护理。在整个外科领域，需要创造一种相互合作的氛围，感染预防和控制、抗菌药物管理和正确的手术方法都至关重要，并且应得到合理的组织。在这种情况下，外科医生的直接领导力是至关重要的，他们直接对病人的安危负责。

**结论**

如果全世界的外科医生都参与到这场全球战斗中来，他们将成为应对这一挑战的关键领导者。否则，他们将成为世界卫生界最严重危机的制造者。

嘿,外科医生!现在你说了算。警惕新的“外科医生的瘟疫”!这是你们参与的时刻，也是你们领导的时刻。现在是行动的时候了!
