# Supplementary material for: Hey surgeons! It is time to lead and be a champion in preventing and managing surgical infections!
Source: World J Emerg Surg. 2020 Apr 19;15:28. doi: 10.1186/s13017-020-00308-1 (PMC7168830; doi:10.1186/s13017-020-00308-1)
Supplement: Supplementary file 3 — Additional file 3:. French translation. [file 13017_2020_308_MOESM3_ESM.docx]

**Additional file 3.** French translation.

By Elie Chouillard and Belinda De Simone.

**Les chirurgiens doivent s’investir davantage dans la prévention et le traitement des infections!**

**Résumé.**

Les mesures de prévention et de gestion du risque infectieux font partie intégrante de la pratique clinique au quotidien et des normes des soins. Cependant, chez les chirurgiens, certaines mesures sont parfois négligées ou sous-estimées. Pourtant, les chirurgiens ont été à l'avant-garde de la prévention et de la prise en charge des infections. L’exercice chirurgical englobe de nombreux processus de soins qui ont une incidence sur le risque d'infections du site opératoire (ISO), d’où l’importance de leur prévention. Les chirurgiens sont également en première ligne dans la gestion des patients atteints d'autres infections, qui ont souvent besoin d'un contrôle rapide de la source septique et d'une antibiothérapie appropriée. Dans ce contexte, le leadership direct des chirurgiens dans la prévention et la gestion des infections est de la plus haute importance.

**Le défi.**

Dans un livre sur l'histoire d'Ignaz Philipp Semmelweis [1], Sherwin B. Nuland, qualifie la fièvre puerpérale de "peste des médecins", car ces mêmes médecins et étudiants en Médecine, qui ont traité les patientes, ont propagé l'infection par leurs mains. En effet, au milieu du XIX^ème^ siècle, une maladie caractérisée par des douleurs, une atteinte de l’état général et une forte fièvre, appelée " fièvre puerpérale", a littéralement décimé les parturientes à l'hôpital universitaire de Vienne où exerçait Semmelweis. Sans connaître l'existence des bactéries (qui ne seront découvertes que près d’un demi siècle plus tard par Louis Pasteur), il remarqua que la mortalité des patientes pouvait être réduite par le simple lavage des mains des médecins avec une solution de chaux chlorée, avant chaque examen. Les observations de Semmelweis étaient en contradiction avec les avis scientifiques et médicaux établis à l'époque. Il est aujourd'hui reconnu comme le "père de la lutte contre les infections".

Depuis la découverte de la Pénicilline par Alexander Fleming à la fin des années 20s du siècle dernier, les antibiotiques ont révolutionné la Médecine. Après avoir sauvé des millions de patients souffrant d’infections bactériennes chaque année, les antibiotiques ont été utilisés à titre prophylactique pour la prévention de ces mêmes infections. Cependant, les bactéries ont développé une résistance aux antibiotiques, provoquant des infections encore plus graves.

Les médecins, par une utilisation inappropriée des antibiotiques et une prévention inadéquate des infections, contribuent au développement et à la propagation de la résistance aux antimicrobiens (RAM). Dans cette perspective, les infections exacerbées par ce phénomène, peuvent être définies comme le nouveau "*fléau des médecins*".

Dans leur pratique clinique, les chirurgiens sont en première ligne pour prévenir et gérer les infections. Cependant, les mesures de prévention sont souvent négligées ou sous-estimées. La non-maitrise de ces mesures a marginalisé les chirurgiens dans ce combat. Dans de nombreux hôpitaux du monde entier, les chirurgiens ne participent pas aux programmes de gestion des antimicrobiens, bien qu'ils en soient de fréquents prescripteurs, tant à titre prophylactique que thérapeutique. En outre, les chirurgiens ne sont souvent pas impliqués dans les équipes de prévention des infections, alors qu'ils peuvent en être les principaux acteurs, en particulier des infections nosocomiales et du site opératoire.

Nous croyons que les chirurgiens font partie de cette lutte planétaire. Il se doivent d’agir en leaders essentiels pour relever ce défi.

**La menace globale de la RAM.**

L’amélioration de la qualité et de la sécurité des soins dans les hôpitaux nécessite une approche systématique pour lutter contre la RAM, à la fois en traitant les infections et en en pratiquant la prévention [2].

La RAM est devenue l'un des principaux problèmes de santé publique du XXI^ème^ siècle. Elle a entraîné une crise d'ampleur internationale, qui menace concomitamment la pratique médicale, la santé animale et la sécurité alimentaire. La menace de la RAM représente sans doute l’un des plus grands défis de notre temps en matière de sécurité des patients. Il a été largement rapporté que le monde est à l'aube d'une "ère post-antibiotique", avec la croissance de bactéries multi-résistantes ; ce qui laisse présager une médecine moderne de moins en moins capable de traiter ce qui est actuellement considéré comme des infections de routine. La RAM est un phénomène naturel qui se produit au fur et à mesure de l'évolution des bactéries. Cependant, des activités humaines intempestives ont accéléré le rythme auquel les bactéries se développent et propagent leur résistance.

**Initiative mondiale pour lutter contre la RAM.**

La lutte contre la menace croissante de la RAM nécessite une approche intégrée systémique et unifiée de la santé publique, animale et environnementale aux échelles locales, nationales et planétaire (*One Health*). Les antibiotiques utilisés pour traiter diverses maladies infectieuses animales peuvent être similaires à ceux utilisés pour les humains. Les bactéries résistantes qui apparaissent chez l'homme, les animaux ou dans l'environnement peuvent se propager de l'un à l'autre des deux règnes des vivants, aussi bien que d'un pays à l'autre. La RAM ne se limite pas aux frontières géographiques ou zoologiques [2].

Les travailleurs de la santé jouent un rôle central dans la prévention de l'émergence et de la propagation de la RAM. Les patients hospitalisés présentent souvent de multiples facteurs de risque pour l'acquisition de bactéries résistantes. Les services de soins aigus sont des incubateurs pour le développement de la RAM. L'ampleur des soins et la fragilité des patients créent un environnement qui facilite à la fois l'émergence et la transmission d'organismes résistants.

**Utilisation appropriée des antibiotiques.**

L'utilisation appropriée des antibiotiques fait partie intégrante d'une pratique clinique optimale. Les antibiotiques peuvent sauver la vie des patients souffrant d'infections bactériennes. Mais leur mésusage revêt plusieurs aspects, et notamment l’absence d’indication valable, pendant une durée excessive ou sans tenir compte des principes pharmacocinétiques [3-4]. Ceci est largement accepté comme un facteur majeur de certaines infections émergentes (telles que celles induites par le Clostridium difficile), de la sélection de pathogènes résistants chez certains patients ou voire de la propagation de la RAM à l'échelle mondiale. Rappelons dans ce contexte le rôle essentiel du microbiote intestinal dans la genèse et le contrôle de plusieurs maladies aiguës et chroniques, ainsi que sa vulnérabilité aux antibiotiques utilisés à mauvais escient.

**Prévention des infections du site opératoire (ISO).**

En 2017, l'Alliance mondiale contre les infections chirurgicales (the Global Alliance for Infections in Surgery: <https://infectionsinsurgery.org>) a élaboré avec plus de 230 experts de 83 pays différents une déclaration sur l'utilisation appropriée des agents antimicrobiens dans les hôpitaux [2]. Les auteurs ont lié la surexposition aux antibiotiques ainsi que leur sur ou mauvaise utilisation au développement de la RAM, en rappelant les principes fondamentaux du traitement des infections et de l’antibioprophylaxie dans toute la filière chirurgicale.

Mieux vaut prévenir que guérir ; toute infection évitée étant une économie à la fois du traitement per se et de ses potentiels effets néfastes. La prévention des infections peut être rentable et mise en œuvre partout, même lorsque les ressources sont limitées.

La communauté chirurgicale continue d'adopter une approche largement perfectible en matière de prévention et de contrôle des infections. Les patients qui portent des dispositifs médicaux (cathéters vasculaires, sondes urinaires, sonde endo-trachéale, trachéotomie, etc.) ou qui subissent des interventions chirurgicales risquent de contracter des infections nosocomiales ; ce qui majore la morbidité et la mortalité, prolonge la durée du séjour hospitalier et abouti à des actions diagnostiques et thérapeutiques supplémentaires. Les chirurgiens ne sont parfois pas suffisamment conscients de cette réalité, avec une application à minima des recommandations. Ces dernières années, une multitude de recommandations pour la prévention des ISO a été publiée [5-7]. Néanmoins, le respect de ces directives reste globalement décevant.

**Contrôle à la source des infections chirurgicales.**

En cas d'infection chirurgicale, la source de l'infection doit être reconnue et contrôlée, pour l’éliminer et réduire l'inoculum bactérien [8-9]. Les infections intra-abdominales ainsi que les infections des tissus mous sont des situations où le contrôle à la source a davantage d’impact sur le pronostic. Le degré d'urgence du traitement est déterminé par le ou les organes affectés, la vitesse relative à laquelle les symptômes cliniques progressent et la stabilité globale du patient.

**Obstacles à surmonter pour les chirurgiens.**

Les instances médicales et scientifiques reconnaissent que la multi-disciplinarité collaborative est essentielle pour prodiguer les meilleurs soins aux patients, optimiser les résultats tant à l’échelle individuelle qu’en matière de prestation globale de santé publique [10].

Une approche collaborative permet à chaque membre de l'équipe d'apporter son expertise et sa contribution aux soins des patients. Réussir le défi de la prévention et du contrôle des infections implique la création d'une culture de collaboration respectée par tous.

Les chirurgiens sont à l'avant-garde de cette philosophie ; ils sont responsables de nombreux processus de soins affectant l’incidence des ISO et leur prévention. Les chirurgiens sont également en première ligne dans la prise en charge des patients atteints d'autres infections, qui ont souvent besoin d'un contrôle rapide de la source d'une antibiothérapie adéquate. Dans ce contexte, leur leadership dans les efforts multidisciplinaires à cet égard est primordial.

Pour être des leaders, les chirurgiens doivent être conscients que la prévention et la gestion appropriées des infections font partie de leur pratique. Dans les hôpitaux, des déterminants culturels, contextuels et comportementaux influencent la pratique clinique.

L'amélioration des comportements en matière de prévention et de gestion des infections reste un défi. Une série de facteurs tels que l'incertitude diagnostique, la crainte de l’échec, les contraintes financières et organisationnelles peuvent compliquer l'approche des chirurgiens. Cependant, en raison de la dissonance cognitive (reconnaître qu'une action est nécessaire mais ne pas la mettre en œuvre), changer les comportements est un défi.

Il existe généralement trois niveaux principaux goulots d’étranglement entravant la modification du comportement des chirurgiens en la matière ; à savoir, individuel, au sein de la corporation et institutionnel.

Au niveau individuel, les chirurgiens devraient avoir les connaissances, les compétences et les capacités nécessaires pour mettre en œuvre des pratiques efficaces de prévention et de gestion des infections. L'amélioration de leurs connaissances peut influencer leurs perceptions et les motiver à changer de comportement. L'éducation et le développement professionnel continu représentent une composante importante pour une mise en œuvre précise des recommandations. La formation des chirurgiens à la prévention et à la gestion des infections devrait commencer au niveau des premières années des études médicales et être consolidée tout au long de la carrière. Les hôpitaux sont responsables de la formation du personnel soignant. Les techniques d'éducation, telles que les ateliers éducatifs, devraient être mises en œuvre dans chaque hôpital du monde entier en fonction de leurs propres ressources.

**Les chirurgiens, leaders d’une lutte multidisciplinaire contre la RAM.**

L'amélioration des connaissances ne suffit pas et ne permet toujours pas de changer les pratiques. Il faut y adjoindre une éducation interactive et continue, des discussions factuelles, l’élaboration de consensus locaux, des retours d'expérience sur les résultats, etc. Il est important d'identifier un leader d'opinion local pour servir de “locomotive” qui porte le changement en l’accélérant. Les chirurgiens ayant une connaissance satisfaisante des infections chirurgicales peuvent fournir un retour d'information aux prescripteurs et mettre en œuvre des changements dans leur propre sphère d'influence en interagissant directement avec les autres instances institutionnelles impliquées dans la lutte contre les infections.

Enfin, des obstacles organisationnels peuvent entraver la prévention et la gestion des infections. Différentes disciplines sont généralement impliquées dans la prévention et la gestion des infections ; ce qui fait de la collaboration, de la coordination, de la communication, du travail d'équipe et de l'efficacité des soins une composante essentielle du succès. La contribution de la multidisciplinarité à l'amélioration des résultats des soins est factuelle et indiscutable. L'utilisation de cette approche renforce le concept selon lequel chaque discipline apporte une expertise particulière et est responsable de ses contributions respectives aux soins des patients. Dans l'ensemble du spectre chirurgical, cela signifie créer une culture de collaboration dans laquelle la prévention et le contrôle des infections, la gestion des antimicrobiens et une approche chirurgicale adaptée sont de la plus haute importance.

**Conclusions.**

Si les chirurgiens participent à ce combat mondial, ils seront des leaders essentiels pour relever ce défi. Sinon, ils auront contribué à l’exacerbation d’une des pires crises à laquelle la santé mondiale a été confrontée.

C'est à vous de décider. C'est à vous de participer. C’est à vous de diriger. C'est le moment d'agir !
