# Supplementary material for: Hey surgeons! It is time to lead and be a champion in preventing and managing surgical infections!
Source: World J Emerg Surg. 2020 Apr 19;15:28. doi: 10.1186/s13017-020-00308-1 (PMC7168830; doi:10.1186/s13017-020-00308-1)
Supplement: Supplementary file 4 — Additional file 4:. German translation. [file 13017_2020_308_MOESM4_ESM.docx]

**Additional file 4.** German translation.

By Andreas Hecker and Birgit Hecker.

**Hey Chirurgen! Es ist Zeit die Führung zu übernehmen und der Vorreiter in der Prävention und im Management chirurgischer Infektionen zu werden!**

**Abstract.**

Passende Maßnahmen zur Prävention und zum Management von Infektionen sind integrale Bestandteile einer optimalen klinischen Versorgung. Von Chirurgen werden diese Maßnahmen oft missachtet. Dennoch, Chirurgen sind an vorderster Front Infektionen zu verhindern und zu managen. Sie sind verantwortlich für viele Prozesse in der Patientenversorgung, die das Risiko für chirurgische Infektionen beinhalten und spielen so eine Schlüsselrolle in ihrer Prävention. Chirurgen sind oftmals die ersten behandelnden Ärzte infizierter Patienten, welche oft eine rasche Fokussanierung und adäquate Antibiotikatherapie benötigen. So sind sie unmittelbar verantwortlich für das Outcome dieser Patientengruppe. In diesem Zusammenhang ist die Führungsrolle der Chirurgen in Infektionsprophylaxe und –management von höchster Wichtigkeit.

Um den Inhalt dieses Editorials weltweit zu verbreiten, wurde es in 9 verschiedene Sprachen übersetzt (Arabisch, Chinesisch, Französisch, Deutsch, Italienisch, Portugiesisch, Spanisch, Russisch, Türkisch).

**Die Herausforderung.**

In einem Buch des Chirurgen Sherwin B. Nuland über die Historie von Ignaz Philipp Semmelweis [1] bezieht sich der Autor auf das Kindbettfieber als die „Doktorenplage“, weil diejenigen Ärzte und Medizinstudenten, die die Patienten behandelten, die Infektion durch ihre Hände übertrugen. Mitte des 19. Jahrhunderts dezimierte eine durch Schmerzen, allgemeines Krankheitsgefühl und hohes Fieber charakterisierte Erkrankung, bekannt als Kindbettfieber, die Zahl junger Mütter im Wiener Universitätskrankenhaus, wo Dr. Semmelweis tätig war. Ohne von der Existenz von Bakterien zu wissen (entdeckt durch Louis Pasteur in der 2. Hälfte des 19. Jahrhunderts) erkannte er, dass die Sterblichkeitsrate durch Händewaschen mit chlorierter Kalklösung vor jeder Untersuchung reduziert werden konnte. Semmelweis´ Beobachtungen führten zu Konflikten mit den etablierten wissenschaftlichen und medizinischen Lehrmeinungen seiner Zeit. Er ist bekannt als „Vater der Infektionskontrolle“.

Mit der Entdeckung des Penicillins durch Alexander Fleming in den späten 1920er Jahren revolutionierten die Antibiotika die Medizin. Sie haben Millionen von Leben jedes Jahr gerettet und wurden prophylaktisch zur Prävention von Infektionserkrankungen eingesetzt. Dennoch haben Bakterien Antibiotikaresistenzen entwickelt, welche durch ihre Resistenz zu ernsthafteren Infektionen führen. Vor diesem Hintergrund könnten die aktuellen Infektionen als die neue „Doktorenplage“ bezeichnet werden, da die gleichen Ärzte durch unangemessenen Antibiotikaeinsatz und inadäquate Infektionsprophylaxe zur Entwicklung und Ausbreitung von antimikrobiellen Resistenzen (AMR) beitragen.

Praktisch tätige Chirurgen sind maßgebend an der Prävention und dem Infektionsmanagement beteiligt. Dennoch sind gerade unter Chirurgen passende Maßnahmen zur Infektionsprävention unbeachtet. Der Mangel an Bewusstsein für diese Maßnahmen hat Chirurgen in diesem Kampf an den Rand gedrängt. In vielen Krankenhäusern weltweit sind Chirurgen nicht an antimicrobial stewardship-Programmen beteiligt, trotz der Tatsache, dass sie regelmäßig Antibiotika zu Therapie und Prophylaxe verschreiben. Zudem sind Chirurgen oftmals nicht Teil von Infektionspräventionsteams, obwohl sie primär verantwortlich für die Prävention von hospital-acquired infections, insbesondere von chirurgischen Wundinfektionen, sind.

Wir konstatieren: Sollten Chirurgen weltweit an diesem globalen Kampf teilnehmen, so werden sie zu bedeutenden Vorreitern, um sich dieser Herausforderung zu stellen.

Um diese Aussage eines solchen Editorials weltweit zu verbreiten wurde sie in 9 verschiedene Sprachen übersetzt (Arabisch, Chinesisch, Französisch, Deutsch, Italienisch, Portugiesisch, Spanisch, Russisch, Türkisch).

**Die globale Bedrohung durch antimikrobielle Resistenzen (AMRs).**

Eine Verbesserung der Patientensicherheit weltweit erfordert einen systematischen Ansatz im Kampf gegen AMR und in der adäquaten Prävention und Behandlung von Infektionen. Beide gehen Hand in Hand [2]. AMR hat sich zu einem der Hauptprobleme im Gesundheitswesen des 21. Jahrhunderts entwickelt. Sie hat sich zu einer öffentlichen Gesundheitskrise von internationaler Dimension entwickelt, welche zusehends die moderne Patientenversorgung, Tiergesundheit und Nahrungsmittelsicherheit gefährdet. Die Bedrohung durch AMR stellt heute eine der größten Herausforderungen an die Patientensicherheit dar. Es wurde vielfach berichtet, dass die Welt an der Schwelle zur „post-antibiotischen Ära“ stehe, mit einer Zunahme an multidrug-resistant Bakterien, was die Befürchtung nährt, dass die moderne Medizin zunehmend unfähig sein wird, heute als Routineinfektionen angesehene Erkrankungen adäquat zu therapieren. AMR ist ein natürliches Phänomen, welches aufkommt, sobald Bakterien auftreten. Dennoch hat menschliches Handeln die Geschwindigkeit der bakteriellen Resistenzentwicklung deutlich beschleunigt.

**Die globale Initiative zum Kampf gegen AMR.**

Der zunehmenden Bedrohung durch AMR entgegenzuwirken erfordert einen ganzheitlichen und multidisziplinären Ansatz – als One Health bezeichnet – weil diejenigen Antibiotika, die zur Behandlung von verschiedenen Tierinfektionen eingesetzt werden, denjenigen für den humanen Gebrauch gleichen können. Resistente Bakterien aus Mensch, Tier oder Umwelt können sich vom einen zum anderen und von Land zu Land übertragen. AMR wird nicht durch geographische oder zoologische Grenzen limitiert [2]. Berufe des Gesundheitswesens spielen eine zentrale Rolle in der Prävention der AMR-Verbreitung.

Hospitalisierte Patienten haben oftmals multiple Risikofaktoren, um AMR zu erwerben. Ambulanzen und Akutkrankenhäuser sind Inkubatoren für die Entwicklung von AMR. Die Intensität der modernen Pflege und die hochempfängliche Patientenpopulation erschaffen eine Umgebung, die sowohl die Entstehung, als auch die Übertragung resistenter Organismen fördert.

**Adäquater Antibiotikagebrauch.**

Adäquater Antibiotikagebrauch ist integraler Bestandteil einer optimalen klinischen Praxis. Antibiotika können lebensrettend sein, wenn Patienten mit bakteriellen Infektionen behandelt werden. Aber sie werden oftmals inadäquat eingesetzt, besonders wenn sie unnötig indiziert und für einen zu langen Zeitraum oder ohne Berücksichtigung pharmakokinetischer Prinzipien eingesetzt werden [3-4]. Der Antibiotikafehlgebrauch wird weithin als Hauptursache zunehmender Infektionen (wie C. difficile), der Selektion resistenter Pathogene in Patienten und der globalen Zunahme von AMR angesehen. Zusätzlich haben aktuelle Erkenntnisse die Rolle des Darmbioms bei akuten und chronischen Erkrankungen und dessen Anfälligkeit für unpassende Antibiotikatherapie gezeigt.

**Prävention chirurgischer Wundinfektionen (SSI).**

Im Jahr 2017 hat die *Global Alliance for Infections in Surgery* bestehend aus über 230 Experten aus 83 verschiedenen Ländern eine weltweite Erklärung zum adäquaten Einsatz von antimikrobiellen Substanzen in Krankenhäusern abgegeben [1]. In dieser Erklärung betonten die Autoren den Beitrag der Antibiotikaexposition, der Fehlindikation, sowie eines „Zu viel“ an Antibiotika zur Entwicklung von AMR und arbeiteten die entscheidenden Prinzipien einer angemessenen Antibiotikaprophylaxe und –therapie in chirurgischen Therapiepfaden heraus.

Bemühungen HAIs vorzubeugen wurden in der Deklaration nicht besonders herausgestellt, jedoch sind sie von signifikanter Wichtigkeit, um die Antibiotikaexposition zu limitieren.

Prävention ist besser als Behandeln und jede verhinderte Infektion ist eine, die keinerlei Behandlung bedarf. Infektionsprävention kann kosteneffektiv sein und überall implementiert werden, auch im Falle begrenzter Ressourcen. Die chirurgische Gemeinschaft setzt dennoch den leichtfertigen Umgang mit der Prävention und Kontrolle von Infektionen fort. Patienten mit Kathetern (zentrale Venenkatheter, Blasenkatheter, Beatmungsschläuche) oder solche, die chirurgischen Eingriffen unterzogen werden, haben ein hohes Risiko für HAIs. HAIs führen zu signifikant erhöhter Morbidität und Letalität, verlängerter Krankenhausverweildauer und benötigen zusätzliche diagnostische und therapeutische Interventionen. Chirurgen sind weiterhin abgestumpft gegenüber dieser Realität und wenig empfänglich für Aufrufe zur Intervention.

Chirurgische Wundinfektionen sind die häufigsten HAIs im chirurgischen Patientengut. In den letzten Jahren wurden viele Leitlinien zur Prävention chirurgischer Wundinfektionen veröffentlicht [5-7]. Trotz klarer Evidenz ist die Compliance allgemein niedrig.

**Fokussanierung chirurgischer Infektionen.**

Tritt eine chirurgische Infektion auf, sollte der Fokus erkannt und saniert werden. Ob Katheter, Abszess oder Device, alle Maßnahmen sollten unternommen werden, die Infektionsquelle zu beseitigen und das bakterielle Inokulum zu beseitigen [8-9]. Adäquate Fokussanierung ist von größter Wichtigkeit für das Management chirurgischer Infektionen. Intraabdominelle Infektionen, wie auch Weichteilinfektionen sind Orte, an denen die Fokussanierung sehr effektiv durchgeführt werden kann. In diesen Fällen kann sie das Outcome der Patienten deutlich verbessern und prolongierte Antibiotikabehandlungen reduzieren. Als allgemeines Prinzip gilt, dass jeder Fokus schnellstmöglich saniert gehört. Die Dringlichkeit ist dabei durch die Mitbeteiligung von Organsystemen, die Geschwindigkeit der Symptomentwicklung und die vorliegende (In-)Stabilität des Patienten festgelegt.

**Hürden, die Chirurgen zu überwinden haben.**

Führende internationale Organisationen erkennen Zusammenarbeit als essentiell für eine patientenzentrierte, individualisierte und optimierte Gesundheitsversorgung an [10]. Ein gemeinsamer Ansatz erlaubt jedem Teilnehmer seine Expertise einzubringen und verantwortlich für seinen bestimmten Beitrag in der Patientenversorgung zu sein. Die Führung in der Prävention und dem Management chirurgischer Infektionen zu übernehmen erfordert, dass eine Kultur der Zusammenarbeit und Kooperation entsteht, bei der Infektionsprävention und kontrolle, antimicrobial stewardship und korrekte chirurgische Ansätze allesamt erörtert und von allen Teammitgliedern beachtet werden.

Chirurgen stehen in vorderster Linie bei der Infektionsprävention. Sie sind verantwortlich für viele Prozesse im Gesundheitswesen, die ein Risiko für chirurgische Wundinfektionen beinhalten und spielen dementsprechend eine wichtige Rolle in deren Prävention. Gleiches gilt für die Behandlung von Patienten mit Infektionen, die oftmals eine schnelle Fokussanierung und adäquate Antibiotikatherapie benötigen, was direkt mit ihrem Outcome korreliert. In diesem Zusammenhang kann ihre Führungsrolle in multidisziplinären Bemühungen zur Qualitätsverbesserung in der Behandlung chirurgischer Patienten gar nicht hoch genug eingeschätzt werden. Um diese Rolle wahrzunehmen, muss dem Chirurgen die adäquate Prävention und Behandlung von chirurgischen Infektionen bewusst sein.

In Krankenhäusern beeinflussen kulturelle und kontextabhängige Verhaltensweisen die Patientenversorgung. Dieses Verhalten zu verbessern bleibt eine Herausforderung.

Eine ganze Reihe von äußeren Umständen wie die diagnostische Unsicherheit, Angst vor klinischem Versagen, Zeitdruck oder organisatorische Probleme können die chirurgische Infektionsbehandlung verkomplizieren. Dennoch – aufgrund der kognitiven Dissonanz (Erkennen, dass die Handlung nötig ist, aber sie dennoch negieren) – bleibt eine Verhaltensänderung herausfordernd.

Es gibt generell drei Ebenen, die die Verhaltensänderung des Chirurgen gegenüber von Infektionsprävention und –behandlung beeinflussen:

1. Intrapersonelle Ebene
2. Interpersonelle Ebene
3. Institutionelle/Organisatorische Ebene

Auf individueller Ebene sollten Chirurgen das nötige Wissen, die Fähigkeiten und Möglichkeiten haben, eine effektive Infektionsprävention und – behandlung zu implementieren. Durch Wissensvermehrung kann ihre Erkenntnis und ihre Motivation zur Verhaltensänderung positiv beeinflusst werden. Ausbildung und Training repräsentieren eine wichtige Komponente für die Implementierung von Empfehlungen. Ausbildung von Chirurgen in Infektionsprävention und –behandlung sollte bereits im Studium beginnen und durch weiteres Training über die postgraduierten Zeit hinaus fortgesetzt werden. Krankenhäuser sind verantwortlich für die Ausbildung des klinischen Personals. Ausbildungsmaßnahmen wie workshops sollten in jedem Krankenhaus weltweit je nach vorhandenen Ressourcen etabliert werden.

**Chirurgen als Champion eines interdisziplinären Kampfes gegen AMR.**

Zunehmendes Wissen allein könnte nicht ausreichend und effektiv genug sein die klinische Tätigkeit zu verändern, es sei denn, sie ist interaktiv, kontinuierlich und schließt Diskussionen über Evidenz, lokalen Konsens, feedback-Diskussionen, Lernpläne etc. ein. Einen lokalen Meinungsführer als „Champion“ herauszuarbeiten ist wichtig, weil er integrativ und motivierend auf die Verhaltensänderung der Kollegen hinarbeiten kann. Chirurgen mit gutem Wissen über chirurgische Infektionen können verschreibenden Ärzten feedback geben und Änderungen in ihrer eigenen Einflusssphäre erreichen indem sie direkt sowohl mit der antibiotic stewardship group, als auch mit der Infection control group interagieren. Hier hat der Ausschluss von Chirurgen zu hohen Barrieren geführt.

Letztlich können organisatorische Hindernisse die Infektionsprävention und –behandlung beeinflussen. Viele verschiedene Krankenhaus-Fachabteilungen sind typischerweise bei der Infektionsprävention und –behandlung beteiligt, was die Kooperation, Koordination, Kommunikation, Team-Zusammenarbeit und effizientes Arbeiten zu essentiellen Elementen des Erfolgs. Es gibt eine große Evidenz, dass effektives Teamwork im Gesundheitswesen zu verbessertem Outcome führt. Dieser Ansatz führt zu dem Konzept, dass jede Fachdisziplin eine bestimmte Expertise einbringt und für ihre spezifischen Beiträge zur Patientenversorgung Verantwortung übernimmt. Quer durch das chirurgische Spektrum sollte eine Kultur der Kooperation entstehen, in der Infektionsprävention und –behandlung, antimicrobial stewardship und die richtigen chirurgischen Ansätze von höchster Wichtigkeit sind und entsprechend korrekt koordiniert werden müssen. In diesem Zusammenhang müssen Chirurgen aus der direkten Patientenversorgung die Führungsrolle übernehmen!

**Schlussfolgerungen.**

Wenn Chirurgen weltweit diesen globalen Kampf annehmen, werden sie zur Speerspitze dieser Herausforderung! Andernfalls tragen sie zur schlimmsten Krise bei, der die Welt gegenübersteht. Hey Chirurgen! Ihr seid dran! Es ist Zeit mitzumachen und Verantwortung zu übernehmen. Es ist Zeit zu handeln!
