# Supplementary material for: Hey surgeons! It is time to lead and be a champion in preventing and managing surgical infections!
Source: World J Emerg Surg. 2020 Apr 19;15:28. doi: 10.1186/s13017-020-00308-1 (PMC7168830; doi:10.1186/s13017-020-00308-1)
Supplement: Supplementary file 5 — Additional file 5:. Italian translation. [file 13017_2020_308_MOESM5_ESM.docx]

**Additional file 5.** Italian translation.

By Davide Luppi and Gennaro Perrone.

**Hey chirurghi! E’ tempo di essere leader e campioni nel prevenire e nel gestire le infezioni chirurgiche!**

**Abstract.**

Misure appropriate di prevenzione e gestione delle infezioni devono far parte integrante della buona pratica clinica e degli standard di cura. Tra i chirurghi, queste misure sono spesso trascurate o ritenute di scarsa rilevanza nella loro pratica quotidiana, quando in realtà proprio loro dovrebbero essere i primi all’avanguardia nella prevenzione e nella gestione delle infezioni. I chirurghi sono (ndr: devono essere) responsabili della maggior parte dei processi di salute pubblica che impattano sul rischio di infezione del sito chirurgico, e giocano pertanto un ruolo chiave nella prevenzione delle infezioni; sono allo stesso tempo in prima linea nella gestione dei pazienti che hanno già un’infezione conclamata, quindi chiamati spesso a risolvere la fonte dell’infezione (source control) e contestualmente a decidere la terapia antibiotica più adeguata, determinandone il risultato.

Per questo è indispensabile che la figura del chirurgo sia di leadership nella gestione e nella prevenzione delle infezioni.

**La sfida.**

Nel libro che racconta la vita di Philipp Semmelweis [1], il chirurgo Sherwin B. Nuland descrive la febbre puerperale come la vera “piaga del medico”; erano infatti proprio i medici e gli studenti che si occupavano dei pazienti (ndr: e delle autopsie), a diffondere e trasmettere “con le loro mani” le infezioni alle puerpere. Per questo a metà del 19° secolo, la cosiddetta “febbre puerperale”, caratterizzata da dolore, malessere generale e febbre, decimò letteralmente molte delle puerpere ricoverate all’ospedale universitario di Vienna dove lavorava il Dott. Semmelweis. Fu lui, senza conoscere ancora l’esistenza dei batteri (scoperti come sappiamo da Louis Pasteur solo nella seconda metà del 19° secolo), a capire che bastava lavarsi le mani con soluzione di cloruro di calce prima di ogni visita alle pazienti, per ridurre significativamente il tasso di mortalità. Le sue osservazioni inevitabilmente entrarono in contrasto con l’opinione medico-scientifica in vigore in quell’epoca. Pur tuttavia (ndr: e per fortuna) viene ancora oggi ricordato come il “padre del controllo delle infezioni”.

Dalla scoperta della Penicillina da parte di Alexander Fleming alla fine degli anni ’20 del 900, gli antibiotici hanno rivoluzionato il campo della medicina. Grazie ad essi è stato possibile salvare milioni di vite ogni anno, arrivando ad utilizzarli anche nella prevenzione delle infezioni. Da allora i batteri hanno però trovato il modo di sviluppare resistenza agli antibiotici, causando infezioni che temiamo sempre di più, fino a considerarle la vera nuova “piaga del medico”; sebbene siano proprio i medici, a causa dell’uso inappropriato degli antibiotici e la scarsa importanza che danno alla prevenzione delle infezioni, a contribuire allo sviluppo e alla diffusione delle resistenze antimicrobiche (AMR).

I chirurghi nella loro pratica clinica quotidiana possono, devono, occuparsi di prevenzione e gestione delle infezioni; anche se sappiamo bene quanto in realtà proprio tra i chirurghi siano spesso ignorate o non applicate le misure di prevenzione. Questa mancanza d’attenzione nei confronti dei protocolli, ha inevitabilmente marginalizzato il chirurgo dalla lotta alle infezioni. In molti ospedali di tutto il mondo, i chirurghi non sono coinvolti nei programmi di stewardship, nonostante siano tra i più grandi utilizzatori di antibiotici, sia nella profilassi, sia nella terapia postoperatoria. Inoltre, spesso non sono nemmeno coinvolti nei gruppi multidisciplinari che si occupano di prevenzione, seppure siano proprio loro i primi a doversi occupare di prevenzione delle infezioni nosocomiali, ed in modo particolare delle infezioni del sito chirurgico. Se i chirurghi di tutto il mondo partecipassero a questa “guerra globale”, potrebbero addirittura essi stessi essere leader e farsi promotori di questa sfida.

**La minaccia globale dell’antibiotico resistenza.**

Al giorno d’oggi per migliorare la sicurezza dei pazienti negli ospedali di tutto il mondo, occorre seguire un approccio sistematico per combattere l’antibiotico resistenza e per prevenire e trattare le infezioni nel modo appropriato. Le due cose devono andare mano nella mano (ndr: o a braccetto) [2].

L’AMR è al momento uno dei principali problemi di salute pubblica del ventunesimo secolo, con ricadute di proporzioni internazionali sia a livello di medicina moderna, che di salute degli animali (impatto sugli allevamenti), arrivando ad interessare la sicurezza alimentare. La minaccia per l’uomo dell’AMR rappresenta indiscutibilmente la più grande sfida dei nostri tempi. E’ ormai certo che il mondo sia giunto nel pieno dell’“era post-antibiotica”; l’aumento nell’incidenza delle infezioni da batteri multiresistenti porta a pensare che la medicina moderna sarà sempre più in difficoltà nel trattare infezioni un tempo facilmente curabili.

L’AMR è un fenomeno naturale che si verifica quando un batterio si evolve; le attività umane non hanno fatto altro che accelerare il ritmo con cui i batteri sviluppano e quindi propagano le resistenze.

**L’iniziativa globale per combattere l’AMR.**

Affrontare adeguatamente l’emergente minaccia dell’AMR richiede un approccio olistico e multidisciplinare - da qui il concetto di One Health, per cui la salute delle persone è direttamente collegata a quella degli animali e dell’ambiente - perché gli antibiotici utilizzati per trattare varie infezioni negli animali possono essere gli stessi utilizzati per l’uomo. I batteri resistenti che si scoprono negli animali, nell’uomo o nell’ambiente, possono passare rispettivamente dall’uno all’altro, e addirittura da una nazione all’altra. L’AMR non ha confini geografici, né tanto meno zoologici [2]; per questo gli operatori sanitari hanno un ruolo centrale nel prevenirne l’insorgenza e la diffusione.

**L’uso appropriato degli antibiotici.**

L’uso appropriato degli antibiotici deve far parte della buona pratica clinica. Gli antibiotici possono salvare la vita se usati adeguatamente. Il problema è che spesso sono usati in modo inopportuno, soprattutto quando non necessari, quando utilizzati per troppi giorni senza motivo, o senza che se ne conosca la farmacocinetica [3-4]. Il loro abuso è considerato uno dei principali responsabili dell’insorgenza di alcune infezioni (ad esempio *Clostridium* difficile), della selezione di patogeni resistenti nei singoli pazienti, e più in generale del continuo sviluppo di AMR a livello globale.

Pare inoltre da recenti studi che il bioma intestinale sia direttamente coinvolto in varie affezioni acute e croniche, e quindi anch’esso vulnerabile agli antibiotici utilizzati in modo inappropriato.

**La prevenzione delle infezioni del sito chirurgico (SSI).**

Nel 2017 la *Global Alliance for Infections in Surgery* ha deciso di condividere con più di 230 esperti da 83 differenti paesi la dichiarazione globale sull’utilizzo appropriato degli agenti antimicrobici negli ospedali di tutto il mondo [1]. All’interno di questa dichiarazione, gli autori hanno sottolineato come l’esposizione agli antibiotici, il loro abuso, ed utilizzo sconsiderato, abbia contribuito allo sviluppo di ABR e hanno quindi indicato i principi fondamentali della profilassi antibiotica e della terapia antibiotica appropriata da applicare lungo tutto il percorso chirurgico.

Nella dichiarazione non si è parlato in modo specifico di come lavorare sulla prevenzione delle infezioni nosocomiali (HAIs), ma sforzi anche in questo senso devono essere fatti per limitare l’esposizione agli antibiotici.

Prevenire è meglio che curare; evitare l’insorgenza di un’infezione significa evitare di utilizzare un antibiotico. Per questo la prevenzione delle infezioni può avere un impatto sui costi, e su di essa si può, si deve lavorare dappertutto, anche nei contesti a risorse limitate.

Il mondo della chirurgia continua ad essere arrogante nel suo approccio alla prevenzione e al controllo delle infezioni. I pazienti in trattamento con dispositivi medicali (cateteri venosi centrali, cateteri vescicali, ventilatori meccanici) o che devono essere sottoposti a procedure chirurgiche sono i più esposti alle infezioni nosocomiali. Le HAIs portano ad un aumento della morbilità e della mortalità, della durata della degenza, e richiedono maggiori interventi diagnostico-terapeutici. Tra queste infezioni, quelle del sito chirurgico (SSIs) sono le più comuni nei pazienti sottoposti a chirurgia.

Negli ultimi anni sono state pubblicate varie linee guida sulla prevenzione delle infezioni del sito chirurgico [5-7]; ciò nonostante l’adeguamento a quanto da esse indicato è universalmente scarso.

**Controllo della fonte nelle infezioni chirurgiche.**

Quando si verifica un'infezione chirurgica, la fonte di infezione deve essere riconosciuta e controllata. Che si tratti di un catetere, di un ascesso o di un dispositivo, devono essere prese tutte le misure per eliminare la fonte e ridurre l'inoculo batterico [8-9]. Nella gestione delle infezioni chirurgiche, un controllo adeguato delle fonti è di massima importanza. Le infezioni intra-addominali, insieme alle infezioni dei tessuti molli, sono i siti in cui il controllo della fonte ha un impatto maggiore. In questi contesti, un adeguato controllo della fonte può migliorare l'outcome dei pazienti e ridurre i cicli prolungati di terapia antibiotica. Come principio generale, ogni fonte accertata di infezione dovrebbe essere controllata al più presto. Il livello di urgenza del trattamento è determinato dagli organi interessati, dalla relativa velocità di progressione dei sintomi clinici e dalla sottostante stabilità fisiologica del paziente.

**Ostacoli da superare per i chirurghi.**

Le principali organizzazioni internazionali riconoscono che la collaborazione è essenziale per fornire cure adeguate e soddisfare le esigenze dei pazienti, ottimizzare i risultati di salute individuali e l’erogazione complessiva di assistenza sanitaria [10].

Un approccio collaborativo consente a ciascun membro del team di fornire esperienza e di essere responsabile dei rispettivi contributi dati alla cura del paziente. Per essere un campione nella prevenzione e nella gestione delle infezioni di tutto lo spettro chirurgico, occorre la creazione di una cultura di collaborazione in cui la prevenzione ed il controllo delle infezioni, la gestione antimicrobica ed il corretto approccio chirurgico siano presi in considerazione e rispettati da tutti i membri del team.

I chirurghi sono in prima linea nella prevenzione delle infezioni. Sono responsabili di molti processi sanitari che incidono sul rischio di SSI e svolgono un ruolo importante nella loro prevenzione. I chirurghi sono inoltre in prima linea nella gestione dei pazienti con infezioni che spesso necessitano un rapido controllo della fonte e un'adeguata terapia antibiotica, pertanto diretti responsabili del loro esito. In questo contesto è fondamentale la loro leadership negli sforzi multidisciplinari per migliorare la qualità della gestione del paziente chirurgico. Per essere leader, i chirurghi devono essere consapevoli che l’appropriata prevenzione e gestione delle infezioni nell’ambito di tutto lo spettro chirurgico sono parte integrante delle migliori pratiche.

Negli ospedali fattori culturali, legati al contesto di lavoro e ai comportamenti che si adottano, influenzano la pratica clinica. Il miglioramento del comportamento nella prevenzione e gestione delle infezioni rimane una sfida.

Elementi come l'incertezza diagnostica, la paura del fallimento clinico, le tempistiche o il contesto organizzativo possono complicare l'approccio del chirurgo alle infezioni. Tuttavia, per la dissonanza cognitiva (riconoscere che un'azione è necessaria ma non attuarla), cambiare comportamento rimane ancora una sfida.

Esistono generalmente tre livelli primari che possono influenzare il cambiamento del comportamento dei chirurghi nella prevenzione e nella gestione delle infezioni. Questi includono:

1) Livello intrapersonale,

2) Livello interpersonale

3) Livello istituzionale o organizzativo.

A livello individuale, i chirurghi dovrebbero avere le conoscenze, gli skills e le capacità necessarie per implementare pratiche efficaci di prevenzione e gestione delle infezioni. Il miglioramento delle loro conoscenze può influenzare le loro percezioni e motivarli a cambiare comportamento. L'educazione e la formazione rappresentano una componente importante per un'attuazione accurata delle raccomandazioni. L'istruzione dei chirurghi nella prevenzione e nella gestione delle infezioni dovrebbe iniziare a livello universitario ed essere consolidata con un'ulteriore formazione durante gli anni post-laurea. Gli ospedali sono responsabili dell'educazione del personale clinico. Le tecniche educative, come i seminari didattici, dovrebbero essere implementate in ogni ospedale del mondo, secondo le proprie risorse.

**I chirurghi come leader e campioni di un gruppo interdisciplinare per combattere la resistenza antimicrobica.**

Tuttavia, aumentare la sola conoscenza potrebbe non essere sufficiente e potrebbe non essere efficace nel cambiare la pratica, a meno che l'istruzione non sia interattiva e continua ed includa discussioni su evidenze cliniche, consenso locale, feedback sulle prestazioni (da parte dei pari), elaborazione di piani di apprendimento personali e di gruppo, ecc. Identificare un opinion leader locale che funga da campione è importante perché il "campione" può integrare le migliori pratiche cliniche e spingere i propri colleghi a cambiare i comportamenti. I chirurghi con una conoscenza soddisfacente delle infezioni chirurgiche possono fornire feedback ai medici prescrittori e implementare il cambiamento all'interno della propria sfera di influenza, interagendo direttamente con il gruppo che si occupa della gestione antimicrobica e con il gruppo responsabile del controllo delle infezioni. I chirurghi esclusi hanno aumentato gli ostacoli all'applicazione delle migliori pratiche cliniche.

Infine, gli ostacoli organizzativi possono influenzare la prevenzione e la gestione delle infezioni. Molte discipline ospedaliere sono generalmente coinvolte nella prevenzione e nella gestione delle infezioni, rendendo la collaborazione, il coordinamento, la comunicazione, il lavoro di squadra e l'assistenza efficiente una componente essenziale del successo. Esistono ora prove sostanziali del fatto che un efficace lavoro di gruppo nell'assistenza sanitaria contribuisca a risultati migliori. L'uso di questo approccio rafforza l’idea che ogni disciplina apporti competenze particolari e sia responsabile dei rispettivi contributi alla cura del paziente. All’interno dell’ambito chirurgico, questo significa creare una cultura di collaborazione in cui la prevenzione e il controllo delle infezioni, la gestione antimicrobica e il corretto approccio chirurgico siano tutti della massima importanza e adeguatamente coordinati. In questo contesto, la leadership diretta dei chirurghi, che sono direttamente responsabili dei loro pazienti, è della massima importanza.

**Conclusioni.**

Se i chirurghi di tutto il mondo parteciperanno a questa lotta globale, saranno leader chiave nell'affrontare questa sfida. Altrimenti, contribuiranno alla peggiore crisi che la salute mondiale abbia affrontato.

Ehi chirurghi! Questa è la vostra chiamata! È tempo di partecipare e di guidare. Adesso è il momento di agire!
