# Supplementary material for: Hey surgeons! It is time to lead and be a champion in preventing and managing surgical infections!
Source: World J Emerg Surg. 2020 Apr 19;15:28. doi: 10.1186/s13017-020-00308-1 (PMC7168830; doi:10.1186/s13017-020-00308-1)
Supplement: Supplementary file 6 — Additional file 6:. Portuguese translation. [file 13017_2020_308_MOESM6_ESM.docx]

**Additional file 6.** Portuguese translation.

By Felipe Couto Gomes, Rafael De Oliveira Nascimento, and Carlos A. Gomes.

**Olá cirurgiões! É hora de liderar e ser campeão na prevenção e tratamento das infecções cirúrgicas!**

**Resumo.**

As medidas de prevenção e tratamento das infecções são componentes essenciais de ótima prática médica, em especial quando respeitam diretrizes padronizadas e o princípio do atendimento integral ao paciente. Todavia, essas recomendações são, em geral, negligenciadas e, portanto, representam ponto de grande preocupação. Como sabemos, os cirurgiões encontram-se na linha de frente, tanto na prevenção quanto no tratamento das infecções; são responsáveis por muitos dos processos envolvidos na assistência médica, que têm impacto direto no risco de infecções do sítio cirúrgico. Assim, são de fundamental importância na prevenção delas, além de constituir os principais agentes no seu tratamento. São responsáveis pela mandatória intervenção precoce para controle do foco e na escolha da terapia antibiótica apropriada, que em última instância impactará seus resultados. Nesse contexto, a liderança direta dos cirurgiões na prevenção e controle de infecções é de extrema importância.

**O desafio.**

No livro, do cirurgião Sherwin B. Nuland, sobre a história de Ignaz Philipp Semmelweis [1], o autor se refere à febre puerperal como a “praga dos médicos”, porque esses, junto com estudantes de medicina, foram responsáveis por disseminar infecção, carreada por meio de suas mãos. Em meados do século XIX, uma doença caracterizada por dor, mal-estar geral e febre alta, conhecida como "febre puerperal", literalmente dizimou puérperas do hospital da Universidade de Viena, local no qual trabalhava o Dr. Semmelweis. Ele, sem saber da existência das bactérias (descoberta por Louis Pasteur apenas na segunda metade do século XIX), entendeu que a taxa de mortalidade poderia ser reduzida, pela simples lavagem das mãos dos médicos assistentes com solução de cal clorada antes de cada exame. As observações de Semmelweis entraram em conflito com opiniões científicas e médicas estabelecidas à época. Ele é hoje conhecido como o "pai do controle de infecções".

Os antibióticos revolucionaram a medicina desde a descoberta da penicilina por Alexander Fleming no final da década de 1920. Desde então, esses antimicrobianos tem poupado milhões de vidas a cada ano, sendo também utilizados na profilaxia e prevenção de doenças infecciosas. No entanto, ao longo dos anos, as bactérias desenvolveram resistência aos antibióticos, causando infecções cada vez mais graves devido ao desenvolvimento de cepas resistentes.

Nesse aspecto, as atuais infecções podem ser definidas como a nova "praga do médico", uma vez que os mesmos, por meio do uso inapropriado deles, seja na terapêutica quanto na profilaxia, estão contribuindo para o desenvolvimento e a disseminação da resistência antimicrobiana (RAM) e infecções mais graves.

Assim, os cirurgiões têm direta responsabilidade na prevenção e tratamento das infecções. No entanto, medidas gerais e específicas de prevenção, estão sendo negligenciadas e colocoram estes profissionais à margem dessa luta. Por outro lado, por todo o mundo, os cirurgiões deveriam se envolver em programas de governância hospitalar na prescrição de antimicrobianos, pois são prescritores frequentes. Além disso, em geral, os cirurgiões não participam de equipes multidisciplares que se dedicam à prevenção de infecções, mesmo sendo os principais agentes ​​executores de atos médicos, responsáveis por infecções adquiridas em hospitais, sobretudo as infecções de sítios cirúrgicos.

Nós admitimos que, se cirurgiões de todo o mundo participarem dessa luta global, eles serão líderes no enfrentamento desse desafio.

**A ameaça global da RAM.**

Melhorar a segurança do paciente no ambiente hospitalar, no contexto mundial, exige abordagem sistemática e sistematizada de combate a RAM, destacando com importância similar os adequados processos envolvidos na prevenção e tratamento das infecções [2].
A RAM surgiu como um dos principais problemas de Saúde Pública do século XXI. Propiciou crise de proporções internacionais, que ameaça a prática da moderna medicina, saúde animal e segurança alimentar. A ameaça da RAM destaca, sem dúvida, como o maior desafio de segurança do paciente do nosso tempo. Tem sido amplamente divulgado que o mundo está à beira de uma 'era pós-antibiótica', com o surgimento de bactérias multirresistentes, elevando a perspectiva que a medicina moderna será cada vez menos capaz de tratar infecções consideradas hoje habituais. A RAM é fenômeno natural que ocorre à medida que as bactérias evoluem. No entanto, as atividades humanas aceleraram o ritmo em que as bactérias desenvolvem e propagam a resistência.

**Iniciativa global contra RAM.**

Enfrentar a crescente ameaça da RAM requer abordagem holística e multidisciplinar - conhecida como One Health - pois os antibióticos usados para tratar diferentes doenças infecciosas de animais podem ser semelhantes aos usados em seres humanos. Ao mesmo tempo, as bactérias resistentes que surgem no homem, nos animais ou no meio ambiente, podem se espalhar de um para outro ambiente e entre países. Portanto, a RAM não se limita às fronteiras geográficas ou zoológicas [2]. Assim, os profissionais de saúde desempenham papel central na prevenção do surgimento e disseminação da RAM.
Pacientes hospitalizados em geral apresentam múltiplos fatores de risco para aquisição de RAM. Instituições de cuidados intensinvos, são incubadoras para o desenvolvimento de RAM, assim como a necessidade de cuidados intensivos e as populações com maior susceptibilidade, criam o ambiente que facilita o surgimento e a transmissão de organismos resistentes.

**Uso apropriado dos antibióticos.**

O uso adequado de antibióticos é parte integrante da prática clínica ideal. Os antibióticos podem salvar vidas ao tratar pacientes com infecções bacterianas. Entretanto, são com frequência utilizados de forma inadequada, particularmente quando a indicação é desnecessária, administrados por tempo excessivo ou desconsiderar os princípios farmacocinéticos [3-4]. O uso indevido é amplamente aceito como fator significativo de algumas infecções emergentes (ex. C. difficile), além de seleção de patógenos resistentes em pacientes específicos e o desenvolvimento contínuo da RAM em todo o mundo. Ademais, investigações recentes indicaram o papel crítico do bioma intestinal em doenças agudas e crônicas e a sua vulnerabilidade a antibióticos inadequados.

**Prevenção de infecções de sítio cirúrgico (ISC).**

Em 2017, a Aliança Global para Infecções em Cirurgia compartilhou com mais de 230 especialistas de 83 países, declaração global sobre o uso apropriado de agentes antimicrobianos em hospitais no contexto mundial [1]. Nesta declaração, os autores destacaram a contribuição da exposição aos antibióticos, seu uso indevido e excessivo como fatores contributivos ao desenvolvimento de RAM, além de delinear princípios fundamentais de profilaxia e terapia antibióticas adequadas ao ambiente cirúrgico. Os esforços para prevenir infecções adquiridas em hospitais (IAH) não foram especificamente delineados na declaração, mas são de importância significativa a limitação da exposição a antibióticos.

Prevenir é melhor que remediar, e toda infecção evitada não necessitará de tratamento. A prevenção pode ser custo-efetiva, além de factível de ser implementada em qualquer lugar, mesmo nos locais de recursos limitados.

A comunidade cirúrgica continua a ser descuidada em sua abordagem à prevenção e ao controle de infecções. Pacientes com os dispositivos médicos (cateteres centrais, cateteres urinários, ventiladores mecânicos) ou submetidos a procedimentos operatórios, correm risco de adquirir IAH. As IAHs resultam em morbidez e mortalidade significativas, prolongam a duração da internação e requerem intervenções diagnósticas e terapêuticas adicionais. Os cirurgiões continuam embotados para essa realidade, com resposta limitada aos pedidos de sua interveniência neste processo.
As infecções do sítio cirúrgico (ISC) são as IAHs mais comuns entre os pacientes cirúrgicos. Nos últimos anos, importantes guidelines sobre diretrizes para a prevenção de infecções do sítio cirúrgico foram publicados [5-7]. Apesar de evidências e diretrizes claras para direcionar estratégias de prevenção de ISC, a adesão é universalmente fraca.

**Controle do foco em infecções cirúrgicas.**

O foco da infecção deve ser reconhecido e controlado sempre que infecção cirúrgica ocorrer, seja ela relacionada à cateter, abscesso ou dispositivo. Todas as medidas devem ser tomadas para eliminar a fonte e reduzir o inóculo bacteriano [8-9]. Destaca-se uma vez mais, que o controle adequado do foco é de extrema importância no tratamento de infecções cirúrgicas. As infecções intra-abdominais, em conjunto com infecções de partes moles, são locais nos quais o controle do foco tem impacto fundamental. Nesse contexto, tal abordagem pode melhorar o resultado dos pacientes e reduzir ciclos prolongados de terapia antimocrobiana. Como princípio geral, todos os focos confirmados de infecção devem ser controladas o mais rápido possível. O nível de urgência do tratamento é determinado pelo (s) órgão (s) afetado (s), a velocidade relativa na qual os sintomas clínicos progridem e a estabilidade dos sistemas orgânicos do paciente.

**Desafios a serem superados pelos cirurgiões.**

As principais organizações internacionais reconhecem que a colaboração é essencial para atender às necessidades dos pacientes, assim como otimizar os resultados individuais e a prestação geral de cuidados médicos em saúde [10].
A abordagem colaborativa permite que cada membro, contribua com conhecimentos especializados e responda por suas respectivas atribuições ao atendimento integral ao paciente. Ser campeão na prevenção e tratamento de infecções no âmbito cirúrgico, envolve a criação desta cultura colaborativa, na qual a prevenção e o controle da infecção, a administração de antimicrobianos e a abordagem operatória correta são respeitados por todos os seus membros.

Como se sabe, os cirurgiões estão na linha de frente na prevenção de infecções. Eles são responsáveis ​​por muitos dos processos de assistência à saúde que impactam o risco de infecções de sítios cirúrgicos (ISC). Eles também estão na liderança do tratamento de pacientes com infecções, que geralmente precisam de imediato controle do foco e terapia antimicrobiana adequada sendo, portanto, diretamente responsáveis ​​por seus resultados. Assim sendo, sua liderança nos esforços multidisciplinares para melhorar a qualidade do paciente cirúrgico é fundamental.

Para serem líderes, os cirurgiões devem estar cientes que a prevenção e a abordagem correta de infecções em todo o ambiente cirúrgico são partes integrantes das melhores práticas clínicas.

Nos hospitais, determinantes culturais, contextuais e comportamentais influenciam a prática clínica, de forma que melhorar o comprometimento na prevenção e manejo de infecções continua sendo um desafio.

Uma série de fatores como incerteza diagnóstica, receio de falha clínica, pressão por tempo ou contextos organizacionais, podem dificultar a abordagem dos cirurgiões em relação às infecções. No entanto, devido à dissonância cognitiva (reconhecer que uma ação é necessária, mas não a implementar), torna a mudança de comportamento desafiadora.

Geralmente, existem três níveis principais que podem influenciar a modificação do comportamento dos cirurgiões na prevenção e tratamento de infecções. São eles:

1) Nível intrapessoal,
2) Nível interpessoal
3) Nível institucional ou organizacional

A nível individual, os cirurgiões devem ter o conhecimento, a experiência e as habilidades necessárias para implementar práticas eficazes de prevenção e tratamento de infecções. Aprimorar seus conhecimentos pode influenciar suas percepções e motivá-los a mudar seus comportamentos. Educação e treinamento representam componentes importantes para a implementação precisa das recomendações. A educação dos cirurgiões na prevenção e tratamento de infecções deve começar na graduação e ser consolidada com treinamento adicional ao longo dos anos de pós-graduação. Os hospitais são responsáveis ​​por educar seu corpo clínico. Técnicas de ensino, como workshops, devem ser implementadas em cada hospital em todo o mundo, de acordo com seus próprios recursos.

**Cirurgiões como líderes e campeões em grupo interdisciplinar para combate a RAM.**

A aquisição isolada do conhecimento pode ser ineficiente e ineficaz na mudança de hábitos, a menos que a educação seja interativa e contínua. Para isso, deve incluir discussões sobre evidências, consensos locais, revisões de “*performance”,* elaboração de planos de aprendizado individual e coletivo, etc. Identificar um líder de opinião local para servir como Campeão é importante, porque o "Campeão" pode integrar as melhores práticas clínicas e levar seus colegas a mudar comportamentos. Cirurgiões com conhecimento satisfatório em infecções cirúrgicas podem fornecer feedback aos prescritores e implementar mudanças em sua própria esfera de influência, interagindo diretamente com o grupo de governância hospitalar e o grupo de controle de infecções. Excluir os cirurgiões deste processo, aumentam as barreiras com intuito de aplicar as melhores práticas clínicas.
Finalmente, os obstáculos organizacionais podem influenciar a prevenção e o tratamento de infecções. Diferentes especialidades no contexto hospitalar estão diretamente envolvidas na prevenção e controle de infecções, tornando a colaboração, coordenação, comunicação, assim como o trabalho em equipe e o atendimento eficiente componentes essenciais do sucesso. Atualmente, existem evidências substanciais que o trabalho em equipe é eficaz na assistência à saúde e contribui para melhores resultados. O uso dessa abordagem reforça o conceito que cada especialidade traz consigo conhecimentos específicos e é responsável por suas respectivas contribuições para o atendimento integral ao paciente. Para todo contexto cirúrgico, deve-se criar a cultura de colaboração, na qual prevenção e controle de infecção, administração antimicrobiana e abordagem cirúrgica correta são de extrema importância se adequadamente coordenadas. Assim sendo, a liderança pontual dos cirurgiões, que detém a responsabilidade direta ​​por seus pacientes, é de extrema importância.

**Conclusão.**

Se cirurgiões de todo o mundo participarem dessa luta global, eles serão líderes no enfrentamento desse desafio. Caso contrário, contribuirão para a pior crise que a saúde mundial está enfrentando.
Olá cirurgiões! A decisão é de vocês! É hora de participar e de liderar. Agora é a hora de agir!
