# Supplementary material for: Hey surgeons! It is time to lead and be a champion in preventing and managing surgical infections!
Source: World J Emerg Surg. 2020 Apr 19;15:28. doi: 10.1186/s13017-020-00308-1 (PMC7168830; doi:10.1186/s13017-020-00308-1)
Supplement: Supplementary file 7 — Additional file 7:. Spanish translation [file 13017_2020_308_MOESM7_ESM.docx]

**Additional file 7.** Spanish translation.

By Ramiro Manzano-Nunez.

**Cirujanos! Es tiempo de liderar y ser defensores en la prevención y el manejo de las infecciones en cirugía.**

**Resumen.**

Las medidas apropiadas de prevención y manejo de infecciones son parte integral de la práctica clínica óptima y los estándares de atención. Entre los cirujanos, estas medidas a menudo se pasan por alto. Sin embargo, los cirujanos deben estar en la primera línea de batalla en la prevención y el manejo de las infecciones. Los cirujanos son responsables de muchos de los procesos de atención médica que afectan el riesgo de infecciones del sitio quirúrgico y juegan un papel clave en su prevención. Además, también juegan un papel principal en el manejo de pacientes con infecciones, que a menudo necesitan un control rápido de la fuente y una terapia antibiótica adecuada siendo fundamentales en el desenlace quirúrgico. En este contexto, el liderazgo directo de los cirujanos en la prevención y el manejo de infecciones es de suma importancia.

**El reto.**

En un libro del cirujano Sherwin B. Nuland sobre la historia de Ignaz Philipp Semmelweis [1], el autor se refiere a la fiebre puerperal como la "peste de los médicos", porque los mismos doctores y estudiantes de medicina que trataban a las pacientes difundían la infección a través de sus manos. A mediados del siglo XIX, una enfermedad caracterizada por dolor, malestar general y fiebre alta, conocida como “fiebre puerperal”, literalmente acabó con las madres hospitalizadas en el hospital de la Universidad de Viena donde trabajaba el Dr. Semmelweis. Él, sin conocer la existencia de bacterias (descubiertas por Louis Pasteur en la segunda mitad del siglo XIX), entendió que la tasa de mortalidad podría reducirse mediante el lavado de manos de los médicos con solución de cal clorada antes de cada examen. Las observaciones de Semmelweis entraron en conflicto con las opiniones científicas y médicas establecidas de la época. Ahora se le conoce como el "padre del control de las infecciones".

Comenzando con el descubrimiento de la penicilina por Alexander Fleming a finales de la década de 1920, los antibióticos han revolucionado el campo de la medicina. Han salvado millones de vidas cada año e incluso se han utilizado profilácticamente para la prevención de enfermedades infecciosas. Sin embargo, las bacterias han desarrollado resistencia a los antibióticos, causando infecciones que son más graves porque son cada vez más resistentes a los antibióticos.

Con esta perspectiva, las infecciones actuales pueden definirse como la nueva "peste de los médicos" porque los médicos a través del uso inapropiado de antibióticos y la prevención inadecuada de infecciones están contribuyendo al desarrollo y la propagación de la resistencia a los antimicrobianos (RAM).

Aunque los cirujanos deben estar en la primera línea de batalla en la prevención y el manejo de infecciones, a menudo ignoran las medidas apropiadas de prevención de infecciones. La falta de conocimiento de estas medidas ha marginado a los cirujanos de esta lucha. En muchos hospitales de todo el mundo, los cirujanos no participan en los programas de control de antibióticos (antimicrobial stewardship programs ) a pesar del hecho de que frecuentemente recetan antibióticos tanto para la profilaxis como para la terapia. Además, los cirujanos suelen no participar en los equipos de prevención de infecciones, pero son los principales responsables de prevenir las infecciones adquiridas en el hospital, particularmente las infecciones del sitio quirúrgico.

Creemos que si los cirujanos de todo el mundo participan y toman un papel protagónico en esta lucha global, serán líderes fundamentales para abordar este desafío.

**La amenaza global de la resistencia a los antimicrobianos (RAM).**

Mejorar la seguridad del paciente en los hospitales del mundo moderno requiere un enfoque sistemático para combatir la RAM y prevenir y tratar las infecciones de manera adecuada. Los dos van de la mano [2]. La RAM se ha convertido en uno de los principales problemas de salud pública del siglo XXI. Esto ha resultado en una crisis de salud pública de proporciones internacionales que amenaza la práctica de la medicina moderna, la salud animal y la seguridad alimentaria. La amenaza de la RAM representa posiblemente el mayor desafío para la seguridad del paciente de nuestro tiempo. Se ha demostrado consistentemente que el mundo está en la cúspide de una "era post-antibiótica". Lo anterior va de la mano del crecimiento de bacterias resistentes a múltiples fármacos que aumentan la posibilidad de que la medicina moderna sea cada vez menos efectiva a la hora de tratar lo que actualmente se consideran infecciones de rutina. La RAM es un fenómeno natural que ocurre a medida que las bacterias evolucionan. Sin embargo, las actividades humanas han acelerado el ritmo al que se desarrollan las bacterias y se propaga la resistencia.

**Uso apropiado de antibióticos.**

El uso apropiado de antibióticos es una parte integral de la práctica clínica óptima. Los antibióticos pueden salvar vidas al tratar pacientes con infecciones bacterianas, pero a menudo se usan de manera inapropiada, específicamente cuando no es necesario o cuando se administran por una duración excesiva o sin tener en cuenta los principios farmacocinéticos [3 - 4]. El uso indebido de antibióticos es ampliamente reconocido como uno de los principales promotores de algunas infecciones emergentes (como C. difficile), la selección de patógenos resistentes y el desarrollo continuo de RAM a nivel mundial. Además, investigaciones recientes han señalado el papel crítico de la microbiota intestinal en las enfermedades agudas y crónicas, y la vulnerabilidad de esta a los antibióticos inapropiados.

**Prevención de las infecciones del sitio operatorio (ISO).**

En 2017, la Alianza Global para Infecciones en Cirugía junto con más de 230 expertos de 83 países publicaron una declaración global sobre el uso apropiado de antibióticos en hospitales de todo el mundo [1]. Dentro de esta declaración, los autores destacaron la contribución de la exposición a los antibióticos, el uso indebido y el uso excesivo para el desarrollo de la RAM y describieron los principios fundamentales de la profilaxis y la terapia con antibióticos apropiados durante la atención quirúrgica.

Las intervenciones y esfuerzos para prevenir las infecciones adquiridas en el hospital no se destacaron específicamente en esta declaración, pero son de gran importancia para limitar la exposición a los antibióticos.

Prevenir es mejor que curar y cada infección prevenida se convierte en una que no necesita tratamiento. La prevención de la infección puede ser rentable e implementarse en todas partes, incluso donde los recursos son limitados.

La comunidad quirúrgica sigue siendo arrogante en su enfoque para la prevención y el control de infecciones. Los pacientes con dispositivos médicos (catéteres centrales, sondas urinarias, ventiladores etc.) o que se someten a procedimientos quirúrgicos corren el riesgo de contraer una infección hospitalaria. Las infecciones adquiridas en el hopsital traen consigo una morbilidad y mortalidad significativas, prolongan la duración de la estancia hospitalaria y requieren intervenciones diagnósticas y terapéuticas adicionales. Los cirujanos continúan siendo ciegos a esta realidad y además frecuentemente tienen una respuesta limitada a las solicitudes de intervención.

Las ISO son las infecciones dquiridas en el hospital más comunes entre los pacientes quirúrgicos. En los últimos años se han publicado pautas para la prevención de infecciones del sitio quirúrgico [5 - 7]. A pesar de que la evidencia es clara y las pautas para dirigir las estrategias de prevención de las ISO han sido publicadas previamente, el cumplimiento de estas es pobre.

**Control de la fuente en infecciones quirúrgicas.**

Cuando ocurre una infección quirúrgica, la fuente de infección debe ser reconocida y controlada. Bien sea que esté relacionada con un catéter, un absceso o un dispositivo, se deben tomar todas las medidas pertinentes para eliminar la fuente y reducir el inóculo bacteriano [8-9]. El control apropiado de la fuente es de suma importancia en el manejo de las infecciones quirúrgicas. Las infecciones intraabdominales junto con las infecciones de tejidos blandos son los sitios donde el control de la fuente tiene un mayor impacto en los desenlaces. Un control de apropiado de la fuente puede mejorar el resultado de los pacientes y reducir los ciclos prolongados de terapia con antibióticos. Como principio general, todas las fuentes de infección comprobadas deben controlarse lo antes posible. El nivel de urgencia del tratamiento está determinado por el órgano u órganos afectados, la velocidad relativa a la que progresan los síntomas clínicos y la estabilidad fisiológica del paciente.

**Obstáculos a superar por parte de los cirujanos.**

Las principales organizaciones internacionales reconocen que la colaboración es esencial para proporcionar una atención adecuada, satisfacer las necesidades de los pacientes, optimizar los resultados de salud individuales y mejorar la prestación de la atención médica [10].

Un enfoque colaborativo permite a cada miembro del equipo contribuir con experiencia y ser responsable con sus contribuciones a la atención al paciente. Ser un líder y defensor en la prevención y el tratamiento de las infecciones en cirugía implica la creación de una cultura de colaboración en la que todos los miembros del equipo sean tenidos en cuenta. Además, se deben repetar los principios de la prevención y el control de las infecciones, el uso racional de antibióticos y el enfoque quirúrgico adecuado.

Los cirujanos deben estar en la primera línea de batalla en la prevención de infecciones. Además, son responsables de muchos de los procesos de atención médica que afectan el riesgo de ISO y desempeñan un papel importante en su prevención. Los cirujanos también deben estar en la primera línea de batalla para obtener un control rápido de la fuente y administrar una terapia antibiótica adecuada. En este contexto, su liderazgo en los esfuerzos multidisciplinarios para mejorar la calidad de los desenlaces del paciente quirúrgico es fundamental.

Para ser líderes, los cirujanos deben ser conscientes de que la prevención y el manejo adecuado de las infecciones en cirugía son una parte integral de las mejores prácticas.

En los hospitales, los determinantes culturales, contextuales y conductuales influyen en la práctica clínica. Mejorar el comportamiento en la prevención y el manejo de infecciones sigue siendo un desafío.

Una variedad de factores como la incertidumbre diagnóstica, el miedo al fracaso clínico, la presión del tiempo o los contextos organizacionales pueden complicar el enfoque de los cirujanos hacia las infecciones. Sin embargo, debido a la disonancia cognitiva ( la tensión o desarmonía interna del sistema de ideas), cambiar el comportamiento es un desafío.

En general, existen tres niveles principales que pueden influir en la modificación del comportamiento de los cirujanos, en la prevención y el manejo de infecciones. Éstos incluyen:

1) El nivel intrapersonal,

2) El nivel interpersonal

3) El nivel institucional u organizacional

A nivel individual, los cirujanos deben tener el conocimiento, la destreza y las habilidades necesarias para implementar prácticas efectivas de prevención y manejo de las infecciones. Mejorar su conocimiento puede influir en sus percepciones y motivarlos a cambiar su comportamiento. La educación y la capacitación representan un componente importante para la implementación adecuada de las recomendaciones basadas en la evidencia. La educación de los cirujanos en prevención y manejo de infecciones debe comenzar en el nivel de pregrado y consolidarse con capacitación adicional durante los años de posgrado. Los hospitales son responsables de educar al personal clínico. Las técnicas para la enseñanza, como los talleres educativos, deben implementarse en cada hospital del mundo de acuerdo con sus propios recursos y contextos culturales.

**Los cirujanos como líderes en un grupo interdisciplinario para luchar contra la RAM.**

Aumentar el conocimiento por sí solo puede no ser suficiente ni efectivo para cambiar la práctica, a menos que la educación sea interactiva y continua, incluya discusiones sobre evidencia y consenso local; contenga retroalimentación sobre el desempeño (por parte de los compañeros), como también planes de aprendizaje personales y grupales, entre otros.

Identificar a un experto local para que sirva como líder es importante porque el "lider" puede integrar las mejores prácticas clínicas e impulsar a sus colegas a cambiar sus comportamientos. Los cirujanos con un conocimiento adecuado en infecciones quirúrgicas pueden brindar retroalimentación a los prescriptores e implementar cambios dentro de su propia esfera de influencia, interactuando directamente con el grupo de control de antimicrobianos y el grupo de control de infecciones. Excluir a los cirujanos ha aumentado las barreras para aplicar las mejores prácticas.

Finalmente, los obstáculos organizacionales pueden influir en la prevención y el manejo de infecciones. Muchas disciplinas hospitalarias diferentes suelen participar en la prevención y el manejo de infecciones, lo que hace que la colaboración, la coordinación, la comunicación, el trabajo en equipo y la atención eficiente sean un componente esencial del éxito. Exista una gran cantidad de evidencia que demuestra que el trabajo en equipo durante la atención médica contribuye a mejorar los resultados clínicos. El uso de este enfoque refuerza el concepto de que cada disciplina aporta experiencia particular y es responsable de sus respectivas contribuciones a la atención del paciente. El control de infecciones durante la atención quirúrgica, significa crear una cultura de colaboración en la que la prevención y el control de infecciones, el control del uso de antimicrobianos y el abordaje quirúrgico correcto son de suma importancia y se coordinan adecuadamente. En este contexto, el liderazgo directo de los cirujanos, que son directamente responsables de sus pacientes, es de suma importancia.

**Conclusiones.**

Si los cirujanos de todo el mundo participan en esta lucha global, serán líderes fundamentales para abordar este desafío. De lo contrario, serán contribuyentes a la peor crisis que enfrenta la salud mundial.

Cirujanos! ¡Escuchen este llamado! Es tu momento de participar y tu tiempo de liderar. ¡Ahora es el momento de actuar!
