# Supplementary material for: Hey surgeons! It is time to lead and be a champion in preventing and managing surgical infections!
Source: World J Emerg Surg. 2020 Apr 19;15:28. doi: 10.1186/s13017-020-00308-1 (PMC7168830; doi:10.1186/s13017-020-00308-1)
Supplement: Supplementary file 8 — Additional file 8:. Russian translation. [file 13017_2020_308_MOESM8_ESM.docx]

**Additional file 8.** Russian translation.

By Andrey Litvin.

**Эй, хирурги! Настало время стать лидером в профилактике и лечении хирургических инфекций!**

**Резюме**

Мероприятия по профилактике и лечению инфекций являются неотъемлемой частью оптимальной клинической практики и стандартов лечения. Среди хирургов эти меры часто игнорируются. Тем не менее, хирурги находятся на переднем крае профилактики и лечения инфекций. Хирурги несут ответственность за выполнение большинства мероприятий, которые влияют на риск развития инфекций в области хирургического вмешательства и играют ключевую роль в их профилактике. Хирурги также занимают передовые позиции в лечении этих пациентов, которым часто требуется быстрое устранение источника инфекции и соответствующая антибактериальная терапия, и которые несут непосредственную ответственность за результаты. В этом контексте непосредственное лидерство хирургов в области профилактики и лечения инфекций имеет первостепенное значение.

**Вызов**

В книге хирурга Sherwin B. Nuland о Земмельвейсе [1] автор называет послеродовую лихорадку «чумой врачей», потому что те же самые врачи и студенты-медики, которые лечили пациентов, распространяли инфекцию через свои руки. В середине девятнадцатого века болезнь, характеризующаяся болью, общей слабостью и высокой температурой, известная как «послеродовая лихорадка», буквально уничтожала родильниц, госпитализированных в больницу Венского университета, где работал Земмельвейс. Он, не зная о существовании бактерий (обнаруженных Луи Пастером только во второй половине XIX века), понимал, что смертность может быть снижена путем мытья рук врачей раствором хлорной извести перед каждым обследованием. Наблюдения Земмельвейса сначала противоречили устоявшимся научным и медицинским знаниям того времени. Сейчас он известен как «отец асептики».

Начиная с открытия пенициллина Александром Флемингом в конце 20-х годов прошлого века, антибиотики произвели революцию в области медицины. Они спасали миллионы жизней каждый год и даже использовались для профилактики инфекционных заболеваний.

Однако, как предсказывали простые дарвиновские эволюционные законы, бактерии развили устойчивость к антибиотикам, вызывая инфекции, которые являются более тяжелыми, потому что они становятся все более устойчивыми к антибиотикам.

С этой точки зрения, современные инфекции также могут быть определены как новая «чума врача», потому что те же самые врачи из-за ненадлежащего использования антибиотиков и неадекватной профилактики инфекций способствуют развитию и распространению антимикробной резистентности (АМР).

Хирурги в своей клинической практике находятся на переднем крае профилактики и лечения инфекций. Тем не менее, среди хирургов, соответствующие мероприятия по профилактике инфекции часто игнорируются. Недостаточная осведомленность об этих мерах оттеснила хирургов от этой борьбы. Во многих больницах по всему миру хирурги не участвуют в программах антимикробной помощи, хотя они часто назначают антибиотики как для профилактики, так и для лечения. Кроме того, хирурги часто не участвуют в бригадах по профилактике инфекций, однако они несут основную ответственность за профилактику внутрибольничных инфекций, особенно инфекций в местах хирургического вмешательства.

Мы утверждаем, если хирурги по всему миру примут участие в этой глобальной борьбе, они станут ведущими лидерами в решении этой проблемы.

**Глобальная угроза от антимикробной резистентности (АМР)**

Необходимость повышения безопасности пациентов в современных больницах по всему миру требует системного подхода к борьбе с АМР, а также к надлежащей профилактике и лечению инфекций. Они идут «рука об руку» [2].

AMР стала одной из главных проблем общественного здравоохранения в 21 веке. Это привело к кризису общественного здравоохранения в международном масштабе, который угрожает в целом практике современной медицины, ветеринарии и продовольственной безопасности. Угроза AMР представляет собой одну из самых больших проблем безопасности пациентов в настоящее время. В средствах массовой информации сообщается, что мир находится на пороге «постантибиотической эры», когда рост бактерий с множественной лекарственной устойчивостью повышает вероятность того, что современная медицина будет все в большей степени неспособна лечить то, что в настоящее время считается обычными инфекциями. AMР - это естественное явление, возникающее по мере развития бактерий. Однако человеческая деятельность ускорила темпы развития бактерий и распространения антимикробной устойчивости.

**Глобальная инициатива по борьбе с AMР**

Устранение растущей угрозы AMР требует общего междисциплинарного подхода - называемого One Health (единое здравоохранение), поскольку антибиотики, используемые для лечения различных инфекционных заболеваний в ветеринарии, могут быть аналогичны тем, которые используются в лечении людей. Устойчивые бактерии, возникающие у людей, животных или в окружающей среде, могут повсеместно распространяться между странами. AMР не ограничивается географическими или зоологическими границами [2]. Следовательно, медицинские работники играют основную роль в предотвращении возникновения и распространения AMР.

Госпитализированные пациенты часто имеют несколько факторов риска для приобретения AMР. Больницы скорой помощи из-за интенсивного лечения большого числа тяжелых и ослабленных пациентов, являются инкубаторами для развития АМР.

**Надлежащее использование антибиотиков - цель 1 для хирургов в борьбе против AMР**

Надлежащее использование антибиотиков является неотъемлемой частью оптимальной клинической практики. Антибиотики могут быть спасительными при лечении пациентов с бактериальными инфекциями. Но они часто используются ненадлежащим образом, особенно при отсутствии показаний, слишком длительном их использовании или без учета фармакокинетических принципов [3-4]. Неправильное использование антибиотиков признано одной из основных причин появления новых инфекций (таких как C. difficile), появления устойчивых патогенов у отдельных пациентов, дальнейшего угрожающего распространения AMР во всем мире. Кроме того, недавние исследования показали важную роль биома кишечника в патогенезе различных острых и хронических заболеваний, а также отрицательное влияние на него использующихся не по показаниям антибиотиков.

**Профилактика инфекции в области хирургического вмешательства (ИОХВ) – 2 цель для хирургов по борьбе с AMР**

В 2017 году Глобальный альянс по инфекционным заболеваниям в хирургии поделился с более чем 230 экспертами из 83 разных стран глобальной декларацией о надлежащем использовании противомикробных препаратов в больницах по всему миру [1]. В этой декларации авторы подчеркнули вклад воздействия, злоупотребления и чрезмерного использования антибиотиков в развитие AMР и изложили основные принципы надлежащей антибиотикопрофилактики и терапии в течение хирургического вмешательства.

Мероприятия по предотвращению внутрибольничных инфекций (ВБИ) не были особо отмечены в этой декларации, но они имеют важное значение для ограничения распространения АМР.

Профилактика лучше лечения, так как каждая предотвращенная инфекция не нуждается в последующем лечении. Профилактика инфекции может быть экономически эффективной и должна осуществляться везде, даже в случаях ограниченных ресурсов.

Хирургическое сообщество не в полной мере использует все возможности по профилактике инфекций и борьбе с ними. Пациенты с различными медицинскими устройствами (сосудистые и мочевые катетеры, ИВЛ), в процессе и после выполнения хирургических вмешательств подвергаются риску инфицирования. ИОХВ приводит к значительной дополнительной заболеваемости и смертности, продлевают продолжительность пребывания в стационаре и требуют дополнительных диагностических и лечебных мероприятий.

Инфекции в области хирургического вмешательства (ИОХВ) являются наиболее распространенными среди пациентов с хирургическим заболеванием. В последние годы было опубликовано много общих руководств по профилактике ИОХВ [5-7]. Несмотря на четкие доказательства эффективности этих хорошо разработанных мероприятий по профилактике ИОХВ, соблюдение их является повсеместно недостаточным.

**Раннее устранение источника хирургических инфекций – 3 цель для хирургов в борьбе с АБР**

Хирургический источник инфекции должен быть своевременно распознан и устранен. Независимо от причины инфекции, необходимо принять все меры для устранения источника и уменьшения бактериальной инокуляции [8-9]. Эффективное устранение источника инфекции имеет первостепенное значение в лечении хирургических инфекций. Внутрибрюшные инфекции, гнойные заболевания мягких тканей хорошо подвергаются устранению источника инфекции. У этих больных устранение источника инфекции может улучшить результаты лечения и сократить длительность курсов антибиотикотерапии. Как правило, каждый диагностированный источник инфекции должен устраняться как можно скорее. Срочность хирургического лечения должна определяться локализацией источника инфекции, скоростью прогрессирования клинических проявлений заболевания, тяжестью физиологического состояния пациента.

**Препятствия, которые хирургам необходимо преодолеть**

Ведущие международные организации признают, что необходимо продолжающееся сотрудничество для организации медицинской помощи, которая будет отвечать потребностям пациентов путем оптимизации индивидуальных подходов и общих принципов оказания медицинской помощи [10].

Подход, основанный на сотрудничестве, позволяет каждому члену команды делиться опытом и нести ответственность за свой вклад в лечение пациента. Чтобы стать чемпионом в профилактике и лечении инфекций во всех хирургических областях, необходимо создать культуру сотрудничества, в которой все члены команды учитывают и уважают общие принципы профилактики и лечения инфекций.

Хирурги находятся на переднем крае профилактики инфекций. Хирурги несут ответственность за многие процессы в здравоохранении в целом, которые влияют на риск возникновения ИОХВ, играют важную роль в профилактике инфекционных осложнений. Хирурги также занимают передовые позиции в лечении пациентов с инфекциями, непосредственно отвечают за результаты их хирургического лечения и адекватность антибактериальной терапии. Лидерство хирургов в междисциплинарном сотрудничестве, направленном на улучшение качества хирургического лечения, является очень важным.

Чтобы быть лидерами, хирурги должны знать, что надлежащая профилактика и лечение инфекций во всем их многообразии является неотъемлемой частью профессиональных успехов.

В некоторых больницах культурные, религиозные и поведенческие традиции могут влиять на результаты лечения. Оптимизация мероприятий по профилактике и лечению инфекций остается в ряде случаев еще проблемой.

Целый ряд факторов, таких как неясность диагноза, риск неблагоприятных результатов лечения, нехватка времени или плохие организационные условия, могут замедлить решение хирурга по своевременной профилактике и лечению инфекционных осложнений. Однако из-за когнитивного диссонанса (признавая, что действие необходимо, но не осуществляя его), изменение поведения является сложной задачей.

Как правило, существует три основных уровня, которые могут влиять на изменение поведения хирургов при профилактике и лечении инфекций. Это включает:

1) внутриличностный уровень,

2) межличностный уровень

3) внутрибольничный или организационный уровень.

На индивидуальном уровне хирурги должны обладать необходимыми знаниями, навыками и способностями для внедрения эффективных методов профилактики и лечения инфекций. Улучшение их знаний может повлиять на их восприятие и мотивировать их на изменение поведения. Образование и обучение представляют собой важный компонент для точного выполнения рекомендаций. Обучение хирургов по профилактике и лечению инфекций должно начинаться с уровня бакалавриата и должно быть объединено с дальнейшей подготовкой на протяжении всего последипломного образования. Больницы несут ответственность за обучение медицинского персонала. Обучение в виде учебных семинаров должно применяться в каждой больнице по всему миру в соответствии с их собственными ресурсами.

**Хирурги как лидеры и чемпионы в междисциплинарных группах по борьбе с AMР**

Одних только расширенных знаний может быть недостаточно и они могут оказаться неэффективными для изменения клинической практики. Это не происходит в тех случаях, когда образование является интерактивным и непрерывным, включает дискуссии о фактических данных, местный консенсус, отзывы о результатах (со стороны коллег), составление личных и групповых планов обучения и т.д. Определение местного лидера общественного мнения, который будет являться чемпионом, важно для интеграции лучших клинических знаний и побуждения своих коллег к изменению поведения. Хирурги с хорошими знаниями в области хирургических инфекций могут обеспечивать обратную связь с клиническими фармакологами и осуществлять изменения в пределах своей сферы влияния с организацией взаимодействия с врачами других для применения наилучшей клинической практики.

В конечном итоге, недостатки в организации лечебного процесса могут препятствовать достижению хороших результатов по профилактике и лечению инфекций. Врачи различных специальностей участвуют в профилактике и лечении инфекций, делая сотрудничество, координацию, общение, командную работу и эффективную помощь неотъемлемой частью успеха. В настоящее время имеется много доказательств того, что эффективная командная работа в сфере здравоохранения способствует улучшению результатов. Использование этого подхода усиливает концепцию, согласно которой врачи всех специальностей приносят свой ценный вклад в эффективное лечение пациентов. Среди врачей хирургического профиля это означает создание культуры сотрудничества, в которой профилактика инфекций и борьба с ними, грамотное антимикробное лечение и правильная хирургическая тактика имеют первостепенное значение и должным образом скоординированы. В этом контексте первостепенное значение имеет непосредственное руководство этим процессом со стороны хирургов, которые несут непосредственную ответственность за своих пациентов.

**Выводы**

Если в этой глобальной борьбе примут участие хирурги всего мира, они станут ведущими лидерами в решении этой проблемы. В противном случае они будут способствовать наихудшему кризису, с которым может столкнуться международная система здравоохранения.

Эй, хирурги! Это ваш вызов. Будьте в курсе новой «чумы хирургов»! Это ваше время для принятия участия и ваше время, чтобы возглавить эту работу. Сейчас самое время действовать!
