# Supplementary material for: Hey surgeons! It is time to lead and be a champion in preventing and managing surgical infections!
Source: World J Emerg Surg. 2020 Apr 19;15:28. doi: 10.1186/s13017-020-00308-1 (PMC7168830; doi:10.1186/s13017-020-00308-1)
Supplement: Supplementary file 9 — Additional file 9:. Turkish translation. [file 13017_2020_308_MOESM9_ESM.docx]

**Additional file 9.** Turkisk translation.

By Arda Isik.

**Hey cerrahlar! Cerrahi enfeksiyonları önleme ve yönetmede lider olma ve şampiyon olma zamanı!**

**Özet.**

Enfeksiyonun önlenmesi ve yönetimi için uygun önlemler, optimal klinik uygulama ve bakım standartlarının ayrılmaz bir parçasıdır. Cerrahlar arasında bu önlemler sıklıkla gözden geçirilir. Bununla birlikte, cerrahlar enfeksiyonları önleme ve yönetmede ön plandadır. Cerrahlar, cerrahi alan enfeksiyonları riskini etkileyen ve önlenmelerinde önemli bir rol oynayan sağlık hizmetlerinin çoğundan sorumludur. Cerrahlar, sıklıkla derhal kaynak kontrolü ve uygun antibiyotik tedavisine ihtiyaç duyan ve sonuçlarından doğrudan sorumlu olan enfeksiyonlu hastaların yönetiminde de ön plandadır. Bu bağlamda, enfeksiyonların önlenmesi ve yönetiminde cerrahların doğrudan liderliği son derece önemlidir.

**Meydan okuma.**

Cerrah Sherwin B.Nuland tarafından Ignaz Philipp Semmelweis'in tarihi üzerine yazılan bir kitapta, [1] yazar puerperal ateşi “doktorların vebası” olarak adlandırır, çünkü hastaları tedavi eden doktorlar ve tıp öğrencileri, enfeksiyonu ellerinden geçirir. On dokuzuncu yüzyılın ortalarında, "puerperal ateş" olarak bilinen ağrı, genel halsizlik ve yüksek ateş ile karakterize edilen bir hastalık, Dr. Semmelweis'in çalıştığı Viyana Üniversitesi hastanesinde yatan yeni annelerin çoğunu öldürdü. Dr. Semmelweis bakteri varlığını bilmeden (Louis Pasteur tarafından on dokuzuncu yüzyılın ikinci yarısında keşfedildi), her muayeneden önce doktorların klorlu kireç çözeltisi ile ellerini yıkamasıyla ölüm oranının düşürülebileceğini anladı. Semmelweis'in gözlemleri, o zamanın yerleşik bilimsel ve tıbbi görüşleri ile çelişiyordu. Artık “enfeksiyon kontrolünün babası” olarak biliniyor.

1920'lerin sonlarında Alexander Fleming'in penisilin keşfinden başlayarak, antibiyotikler tıp alanında devrim yarattı. Her yıl milyonlarca hayat kurtardılar ve hatta bulaşıcı hastalıkların önlenmesi için profilaktik olarak kullanıldılar. Bununla birlikte, bakteriler antibiyotiklere karşı direnç geliştirdi ve antibiyotiklere karşı giderek daha dirençli oldukları için daha ciddi enfeksiyonlara neden oldu.

Bu bakış açısıyla, mevcut enfeksiyonlar yeni "doktorun vebası" olarak tanımlanabilir, çünkü aynı doktorlar uygunsuz antibiyotik kullanımı ve yetersiz enfeksiyon önleme yoluyla antimikrobiyal rezistansın (AMR) gelişimine ve yayılmasına katkıda bulunmaktadır.

Cerrahlar klinik uygulamalarında enfeksiyonları önleme ve yönetmede ön plandadır. Bununla birlikte, cerrahlar arasında, enfeksiyonun önlenmesine yönelik uygun önlemler genellikle göz ardı edilir. Cerrahlar bu konuyu gözardı ettikleri için bu tartışmadan uzak kaldılar. Dünyadaki birçok hastanede, cerrahlar hem profilaksi hemde terapi için sık sık antibiyotik reçetesi yazmasına rağmen antimikrobiyal gözetim programlarına dahil değildir. Ayrıca, cerrahlar genellikle enfeksiyon önleme ekiplerine dahil değildir, öncelikle hastane kaynaklı enfeksiyonları, özellikle cerrahi alan enfeksiyonlarını önlemekten sorumludurlar.

Dünyanın dört bir yanındaki cerrahlar bu küresel savaşa katılırlarsa, bu zorluğun üstesinden gelmede çok önemli liderler olacaklarını iddia ediyoruz.

**Küresel AMR tehdidi.**

Dünya çapındaki hastanelerde hasta güvenliğinin iyileştirilmesi için; AMR ile mücadele, enfeksiyonların uygun şekilde önlenmesi ve tedavi edilmesi, beraber bir yaklaşım gerektirir. İkisi iç içedir [2].

AMR, 21. yüzyılın en önemli halk sağlığı sorunlarından biri olarak ortaya çıkmıştır. Bu, modern tıp, hayvan sağlığı ve gıda güvenliği uygulamalarını tehdit eden uluslararası alanda bir halk sağlığı kriziyle sonuçlanmıştır. AMR tehdidi tartışmasız zamanımızın en büyük hasta güvenliği sorunudur. Çoklu ilaca dirençli bakterilerin büyümesi, modern tıbbın şu anda rutin enfeksiyonlar olarak kabul edilenleri tedavi edemeyeceği ihtimalini artıran, dünyanın bir 'antibiyotik sonrası dönemin' zirvesinde olduğu kanısını güçlendirmiştir. AMR, bakteriler geliştikçe ortaya çıkan doğal bir olgudur. Bununla birlikte, insan faaliyetleri, bakterilerin yayılmasını ve direnç geliştirmesini hızlandırmıştır.

**AMR ile mücadele için küresel girişim.**

Artan AMR tehdidinin ele alınması, çeşitli hayvan bulaşıcı hastalıkların tedavisinde kullanılan antibiyotiklerin insanlar için kullanılanlara benzer olabileceği için - Tek Sağlık olarak adlandırılan bütünsel ve multidisipliner bir yaklaşım gerektirir. İnsanlarda, hayvanlarda veya çevrede ortaya çıkan dirençli bakteriler birbirlerine ve bir ülkeden diğerine yayılabilir. AMR coğrafi veya zoolojik alanlarda sınırlı değildir [2]. Sağlık çalışanları AMR'nin ortaya çıkmasını ve yayılmasını önlemede merkezi bir rol oynamaktadır.

Hastaneye yatırılan hastalarda sıklıkla AMR gelişimi için birden fazla risk faktörü vardır. Akut bakım tesisleri, AMR gelişimi için inkübatörlerdir. Bakım yoğunluğu ve oldukça hassas popülasyonlar, dirençli organizmaların ortaya çıkmasını ve bulaşmasını kolaylaştıran bir ortam yaratır.

**Antibiyotiklerin uygun kullanımı.**

Antibiyotiklerin uygun kullanımı, optimal klinik uygulamanın ayrılmaz bir parçasıdır. Bakteriyel enfeksiyonu olan hastaları tedavi ederken antibiyotikler hayat kurtarıcı olabilir. Ancak genellikle uygunsuz, özellikle gereksiz veya aşırı süre veya farmakokinetik prensipler dikkate alınmadan kullanılırlar [3-4]. Antibiyotiklerin gereksiz kullanımı, bazı ortaya çıkan enfeksiyonların (C. difficile gibi) oluşumunda, hastalarda dirençli patojenlerin gelişmesinde ve küresel olarak AMR'nin devam eden gelişimi için büyük bir itici güç olarak kabul edilmektedir. Ek olarak, son araştırmalar bağırsak mikrobiyotasının akut ve kronik hastalıklarda kritik rolünü ve uygunsuz antibiyotiklere karşı savunmasız olduğunu göstermiştir.

**Cerrahi alan enfeksiyonlarının (SSI) önlenmesi.**

2017 yılında Küresel Cerrahi Enfeksiyonlar İttifakı, 83 farklı ülkeden, 230'dan fazla uzmanla, dünya çapında hastanelerde antimikrobiyal ajanların uygun kullanımı hakkında küresel bir bildiri paylaşmıştır [1]. Bu bildiride yazarlar, antibiyotik maruziyeti, gereksiz kullanım ve aşırı kullanımın AMR gelişimine katkısını vurgulamış, cerrahi yol boyunca uygun antibiyotik profilaksisi ve tedavisinin temel prensiplerini ana hatlarıyla belirtmiştir.

Hastane kaynaklı enfeksiyonları (HKE) önlemeye yönelik çabalar bildirimlerde özellikle vurgulanmamıştır, ancak bunlar antibiyotik maruziyetini sınırlamada önemli role sahiptir.

Önleme, tedaviden daha etkilidir ve önlenen her enfeksiyon, tedavi gerektirmeyen bir enfeksiyondur. Enfeksiyonun önlenmesi, kaynakların sınırlı olduğu yerlerde bile uygun maliyetli olarak uygulanabilir.

Cerrahi komite enfeksiyonun önlenmesi ve kontrolüne yaklaşımında daha cesaretli olmaya devam etmektedir. Tıbbi cihazlara (santral kateter, idrar sondaları, ventilatörler) maruziyeti olan veya cerrahi prosedürler geçiren hastalar HKE geçirme riski altındadır. HKE'ler önemli morbidite ve mortalite ile sonuçlanır, hastanede kalış süresini uzatır, ek teşhis ve tedavi müdahalelerini gerektirir. Cerrahlar, müdahale taleplerine sınırlı yanıt vererek bu gerçekliğe aykırı davranmaya devam etmektedirler.

Cerrahi alan enfeksiyonları (SSI'lar) cerrahi hastalar arasında en yaygın HKE'lerdir. Son yıllarda, cerrahi alan enfeksiyonlarının önlenmesi için birçok kapsamlı kılavuzlar yayınlanmıştır [5-7]. SSI’ları önleme stratejilerinin etkinliğini gösteren açık kanıtlara ve klavuzlara rağmen, uyum evrensel olarak zayıftır.

**Cerrahi enfeksiyonlarda kaynak kontrolü.**

Cerrahi enfeksiyon meydana geldiğinde, enfeksiyonun kaynağı tanınmalı ve kontrol edilmelidir. Kateter, apse veya tıbbi cihaz ile ilgili olan/olmayan, kaynağı ortadan kaldırmak ve bakteriyel çoğalmayı azaltmak için tüm önlemler alınmalıdır [8-9]. Cerrahi enfeksiyonların tedavisinde uygun kaynak kontrolü çok önemlidir. Karın içi enfeksiyonlar ve yumuşak doku enfeksiyonları, kaynak kontrolünün etkili olduğu bölgelerdir. Bu ortamlarda uygun bir kaynak kontrolü hastaların sonuçlarını iyileştirebilir ve uzun süreli antibiyotik tedavisi seyrini azaltabilir. Genel bir ilke olarak, doğrulanmış her enfeksiyon kaynağı mümkün olan en kısa sürede kontrol edilmelidir. Tedavinin aciliyeti, etkilenen organ (lar), klinik semptomların ilerleme hızı ve hastanın altta yatan fizyolojik stabilitesi tarafından belirlenir.

**Cerrahlar için aşılması gereken engeller.**

Önde gelen uluslararası kuruluşlar, işbirliğinin, hastaların ihtiyaçlarını karşılamada, bireysel sağlık sonuçlarını optimize etmede ve genel sağlık hizmet sunumunu iyileştirmede, gerekli olduğunu kabul etmektedir [10].

İşbirliğine dayalı bir yaklaşım, ekibin her üyesinin katkıda bulunmasını ve hasta bakımına yaptıkları katkılardan sorumlu olmasını sağlar. Cerrahi spektrumda enfeksiyonları önleme ve yönetmede şampiyon olmak, enfeksiyon önleme ve kontrolun, antimikrobiyal yönetimin ve doğru cerrahi yaklaşımın tüm ekip üyeleri tarafından dikkate alındığı ve saygı duyulduğu bir işbirliği kültürünün oluşturulmasını içerir.

Cerrahlar enfeksiyonları önlemede ön plandadır. Cerrahlar, SSI riskini etkileyen ve bunların önlenmesinde önemli bir rol oynayan sağlık süreçlerinin çoğundan sorumludur. Cerrahlar, derhal kaynak kontrolünün ve uygun antibiyotik tedavisine ihtiyaç duyulan enfeksiyonlu hastaların yönetiminde de ön plandadır. Bu bağlamda, cerrahi hastanın tedavi başarısını artırmak için multidisipliner yaklaşımdaki liderlikleri kritik öneme sahiptir.

Cerrahlar liderlik için, cerrahi spektrumda enfeksiyonların uygun şekilde önlenmesi ve yönetilmesinin, en iyi cerrahi uygulamanın ayrılmaz bir parçası olduğunun farkında olmalıdırlar.

Hastanelerde kültürel, bağlamsal ve davranışsal belirleyiciler klinik uygulamayı etkiler. Enfeksiyonun önlenmesi ve yönetiminde davranışların iyileştirilmesi hala bir zorluktur.

Tanısal belirsizlik, klinik başarısızlık korkusu, zaman baskısı veya organizasyonel problemler gibi bir dizi faktör cerrahların enfeksiyonlara yaklaşımını zorlaştırabilir. Bununla birlikte, bilişsel uyumsuzluk nedeniyle (bir eylemin gerekli olduğunu ancak yerine getirmediğini kabul ederek), davranışları değiştirmek zordur.

Genellikle cerrahların enfeksiyonun önlenmesi ve yönetimi konusundaki davranış değişikliğini etkileyebilecek üç temel seviye vardır. Bunlar:

1) Kişisel seviye,

2) Kişilerarası seviye

3) Kurumsal veya örgütsel düzey

Bireysel düzeyde, cerrahlar etkili enfeksiyon önleme ve yönetimini uygulamak için gerekli bilgi, beceri ve yeteneklere sahip olmalıdır. Bilgilerini geliştirmek, algılarını etkileyebilir ve onları davranışları değiştirmeye motive edebilir. Eğitim ve öğretim, tavsiyelerin doğru uygulanması için önemli bir bileşendir. Enfeksiyonların önlenmesi ve yönetiminde cerrahların eğitimi lisans düzeyinde başlamalı ve lisansüstü yıllar boyunca ileri eğitim ile birleştirilmelidir. Hastaneler klinik personeli eğitmekten sorumludur. Eğitim atölyeleri gibi eğitim teknikleri dünya çapındaki her hastanede kendi kaynaklarına göre uygulanmalıdır.

**AMR ile savaşmak için disiplinlerarası bir grupta lider ve şampiyon olarak cerrahlar.**

Bununla birlikte, eğitimin etkileşimli ve sürekli olmadığı, beraberinde kanıt, yerel konsensüs, performansla ilgili geri bildirimler (akranlar tarafından), kişisel ve grup öğrenme planları yapma vb. ile ilgili tartışmaları içermediği sürece, tek başına artan bilgi yeterli olmayabilir ve değişen uygulamada etkili olmayabilir. “Şampiyon”, en iyi klinik uygulamaları entegre edebileceği ve meslektaşlarını değişen davranışlara yönlendirebileceği için, bir şampiyon olarak görev yapacak yerel bir fikir liderinin belirlenmesi önemlidir. Cerrahi enfeksiyonlarda yeterli bilgiye sahip cerrahlar, reçete yazanlara geri bildirim sağlayabilir ve kendi etki alanlarında değişiklik yaparak hem antimikrobiyal gözetim grubu hem de enfeksiyon kontrol grubu ile doğrudan etkileşime girebilirler. Cerrahları dışlamak, en iyi uygulamanın yapılmasını engeller.

Son olarak, organizasyonel engeller enfeksiyonun önlenmesi ve yönetimini etkileyebilir. Birçok farklı hastane disiplinleri tipik olarak enfeksiyonun önlenmesi ve yönetiminde yer alır ve işbirliğini, koordinasyonu, iletişimi, ekip çalışmasını ve etkin bakımı başarının önemli bir bileşeni yapar. Artık sağlık hizmetlerinde etkili ekip çalışmasının iyileştirilmiş sonuçlara katkıda bulunduğuna dair önemli bir kanıt vardır. Bu yaklaşımı kullanmak, her disiplinin özgün yetkinlik sağladığı ve hasta bakımına katkıdan sorumlu olduğu kavramını güçlendirir. Cerrahi spektrumda, enfeksiyon önleme ve kontrol, antimikrobiyal yönetim ve doğru cerrahi yaklaşım, son derece önemli ve uygun şekilde koordine edilen bir işbirliği kültürü oluşturmak anlamına gelir. Bu bağlamda, hastalarından direk sorumlu olan cerrahların doğrudan liderliği son derece önemlidir.

**Sonuçlar.**

Dünyanın dört bir yanındaki cerrahlar bu küresel savaşa katılırlarsa, bu zorluğun üstesinden gelmede çok önemli liderler olacaklardır. Aksi takdirde, dünya sağlığının yaşadığı en büyük krize sebep olunacaktır.

Hey cerrahlar! Bu senin çağrın! Katılma zamanı ve liderlik zamanı. Şimdi harekete geçme zamanı!
